# Supplementary material for: Identification of tagged glycans with a protein nanopore
Source: Nat Commun. 2023 Mar 28;14:1737. doi: 10.1038/s41467-023-37348-5 (PMC10050315; doi:10.1038/s41467-023-37348-5)
Supplement: Supplementary file 1 — Supplementary Information [file 41467_2023_37348_MOESM1_ESM.pdf]

Supplementary Information for

Identification of tagged glycans with a protein nanopore

Minmin Li[1,2], Yuting Xiong[1,2], Yuchen Cao[1], Chen Zhang[3], Yuting Li[3], Hanwen Ning[4], Fan Liu[1], Han Zhou[1,3], Xiaonong Li[3], Xianlong Ye[3], Yue Pang[5], Jiaming Zhang[4], Xinmiao Liang[1,3]* and Guangyan Qing[1]*

[1]CAS Key Laboratory of Separation Science for Analytical Chemistry, Dalian Institute of Chemical Physics, Chinese Academy of Sciences, Dalian 116023, P. R. China.

[2]Jiangxi Province Key Laboratory of Polymer Micro/Nano Manufacturing and Devices, School of Chemistry, Biology and Materials Science, East China University of Technology, Nanchang 330013, P. R. China.

[3]Jiangxi Provincial Key Laboratory for Pharmacodynamic Material Basis of Traditional Chinese Medicine, Ganjiang Chinese Medicine Innovation Center, Nanchang 330000, P. R. China.

[4]Department of Statistics, Zhongnan University of Econometrics and Law, Wuhan 430073, P. R. China.

[5]College of Life Science, Liaoning Normal University, Dalian 116081, P. R. China.

E-mail: liangxm@dicp.ac.cn; qinggy@dicp.ac.cn.

Supplementary method

Regents and materials

1,2-diphytanoyl-*sn*-glycero-3-phosphocholine (DPhPC, powder, ≥99%) was purchased from Avanti Polar Lipids, Inc. (Alabaster, AL, USA). n-Decane (99%) was purchased from Thermo Fisher Scientific Inc. (Waltham, MA, USA). Trypsin agarose (T1763, buffered aqueous suspension, from bovine pancreas trypsin) and potassium chloride (for molecular biology, ≥99.0%) were purchased from Sigma-Aldrich Co., Ltd. (St. Louis, MO, USA). Tris(hydroxymethyl) aminomethane (Tris, 99%) was purchased from Beijing Solarbio Science & Technology Co., Ltd. (Beijing, China). Ethylenediaminetetraacetic acid disodium salt (EDTA-2Na, ≥99.0%), 1-naphthylamine (99%), 1-aminopyrene (97%), D-lactose monohydrate (98%), D-(+)-maltose monohydrate (98%), 4,4'-diaminodiphenyl ether (98%, Cas No. 101-80-4), and sodium cyanoborohydride ($NaBH_3CN$, 95%) were purchased from Beijing Innochem Science & Technology Co., Ltd. (Beijing, China). 3'-Sialylgalactose sodium salt (3SG, Neu5Acα2–3Gal, Cas No. 83563-61-5, >90.0%), 6'-sialylgalactose sodium salt (6SG, Neu5Acα2–6Gal, Cas No. 35259-23-5, >90.0%), Cytidine-5'-monophospho-*N*-acetylneuraminic acid sodium salt (CMP-Sia, CAS No. 3063-71-6), α2–6-sialyltransferase (Pd26ST, 5.78 U/mL, *E.coli* recombinant from *Photobacterium damsel*), and α2–3-sialyltransferase (PmST1, *E. coli* recombinant from *Pasteurella multocida* (P-1059)) were purchased from Wuhan Tangzhi Pharmaceutical Co., Ltd. (Wuhan, China). 3'-Sialyllactose sodium salt (3SL, Neu5Acα2–3Galβ1–4Glc, >98.0%), 6'-sialyllactose sodium salt (6SL, Neu5Acα2–6Galβ1–4Glc, >98.0%), and Cytidine 5'-monophosphate disodium salt (CMP) were purchased from TCI (Shanghai) Development Co., Ltd. (Shanghai, China). 2'-Fucosyllactose (2FL, Fucα1–2Galβ1–4Glc, >95.0%), 3'-fucosyllactose (3FL, Galβ1–4(Fucα1–3)Glc, >95.0%), Lacto-*N*-neotetraose (LNnT, Galβ1–4GlcNAcβ1–3Galβ1–4Glc, >95.0%), and Lacto-*N*-tetraose (LNT, Galβ1–3GlcNAcβ1–3Galβ1–4Glc, >95.0%) were purchased from Shanghai HuicH Biotech Co., Ltd. (Shanghai, China). Sialylated tetraose type 2 sodium salt (STetra2, Neu5Acα2–3Galβ1–4GlcNAcβ1–3Gal, >85% from NMR), LS-Tetrasaccharide a/Sialyllacto-*N*-tetraose a sodium salt (LSTa, Neu5Acα2–3Galβ1–3GlcNAcβ1–3Galβ1–4Glc, >90% from NMR), Lewis A (LeA) triaose (Galβ1–3(Fucα1–4)GlcNAc), and Lewis X (LeX) triaose (Galβ1–4(Fucα1–3)GlcNAc, >90% from NMR) were purchased from ELICITYL France. LS-Tetrasaccharide b/Sialyllacto-*N*-tetraose b (LSTb, Neu5Acα2–6[Galβ1–3]GlcNAcβ1–3Galβ1–4Glc, >95% from HPLC), LS-Tetrasaccharide c/Sialyllacto-*N*-tetraose c (LSTc, Neu5Acα2–6Galβ1–4GlcNAcβ1–3Galβ1–4Glc, >90% from NMR), and LS-Tetrasaccharide d/Sialyllacto-*N*-tetraose d (LSTd, Neu5Acα2–3Galβ1–4GlcNAcβ1–3Galβ1–4Glc, >90% from NMR) were purchased from Shanghai ZZBIO. CO., LTD. p-Anisidine (99%), 1-(4-aminophenyl)-1,2,2-triphenylethene (97%), D-(+)-cellobiose (98%), and 1-amylamine (98%) were purchased from Shanghai Aladdin Biochemical Technology Co., Ltd. (Shanghai, China).

4-(4-Methoxyphenoxy) aniline (CAS No. 31465-36-8, 97%) was purchased from Ark Pharm, Inc. (Arlington Heights, IL, USA). Other general reagents and solvents were purchased from Sinopharm Chemical Reagent Co., Ltd. (Shanghai, China). All solutions were prepared using ultrapure water (18.2 MΩ·cm^{-1} at 25 °C) from a Milli-Q system. Ag/AgCl electrode was prepared by electroplating from a silver wire (0.5 mm in diameter) as the anode (a platinum wire served as the cathode) in 0.1 M HCl under a constant 1 mA current for 10 minutes.

Synthesis and characterization of 3SL-MB and 6SL-MB

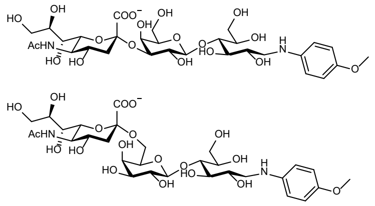

Sialylted trisaccharide (3SL and 6SL) derivatives with the 1-methoxybenzene (MB) tag (*i.e.*, **3SL-MB** and **6SL-MB**) were prepared according to the *general procedure* from 3'-sialyllactose or 6'-sialyllactose (10 mg) with p-anisidine (3.75 mg) in the presence of $NaBH_3CN$ (4.8mg). The gradient elution condition of HPLC separation is 10%–50% elute B in 12 minutes. The collected fraction was concentrated and subjected to freeze drying to obtain the target product in 63% yield (8.4 mg, 0.011 mmol) for 3SL-MB and 72% yield (9.6 mg, 0.013 mmol) for 6SL-MB. Q-TOF-MS *m/z* for 3SL-MB ($C_{30}H_{48}N_2O_{19}$): calcd. $[M+H]^+$ 741.2924, found 741.2918. Analytical HPLC gave retention time (T_R) of 8.212 min over 12 min with 5%–90% elute B, and purity of 99.1%. Q-TOF-MS *m/z* for 6SL-MB ($C_{30}H_{48}N_2O_{19}$): calcd. $[M+H]^+$ 741.2924, found 741.2933. Analytical HPLC gave T_R of 8.157 min over 12 min with 5%–90% elute B, and the purity of 91.8%.

Synthesis and characterization of 3SL-MPB and 6SL-MPB

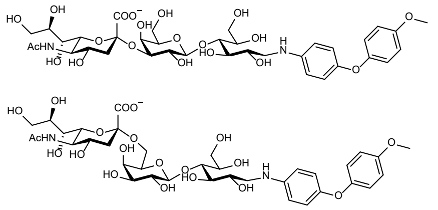

Sialylted trisaccharide (3SL and 6SL) derivatives with the 1-methoxy-4-phenoxybenzene (MPB) tag (*i.e.*, **3SL-MPB** and **6SL-MPB**) were prepared according to the *general procedure* from 3'-sialyllactose or 6'-sialyllactose (10 mg) with 4-(4-methoxyphenoxy) aniline (6.5 mg) in the presence of $NaBH_3CN$ (4.8 mg). The gradient elution condition of HPLC separation is 20%–74% elute B in 12 minutes. The collected fraction was concentrated and subjected to freeze drying to obtain the target product in 67% yield (10 mg, 0.012 mmol) for 3SL-MPB and 72% yield (10.6 mg, 0.013 mmol) for 6SL-MPB. Q-TOF-MS *m/z* for 3SL-MPB ($C_{36}H_{52}N_2O_{20}$): calcd. for $[M+H]^+$ 833.3186, found 833.3273. Analytical HPLC gave T_R of 10.110 min over 12 min with 10%–90% elute B, and purity of 99.7%. Q-TOF-MS *m/z* for 6SL-MPB ($C_{36}H_{52}N_2O_{20}$): calcd. for $[M+H]^+$ 833.3186, found 833.3278. Analytical HPLC gave T_R of 9.891 min over 12 min with 10%–90% elute B, and purity of 99.8%.

Synthesis and characterization of 3SL-NA and 6SL-NA

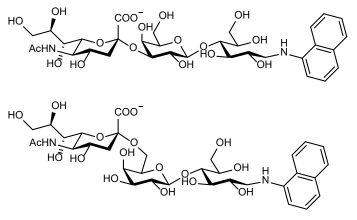

Sialylted trisaccharide (3SL and 6SL) derivatives with the naphthyl (NA) tag (*i.e.*, **3SL-NA** and **6SL-NA**) were prepared according to the *general procedure* from 3'-sialyllactose or 6'-sialyllactose (10 mg) with 1-naphthylamine (4.4 mg) in the presence of $NaBH_3CN$ (4.8 mg). The gradient elution condition of HPLC separation is 20%–60% elute B in 12 minutes. The collected fraction was concentrated and subjected to freeze drying to obtain the target product in 76% yield (10.4 mg, 0.014 mmol) for 3SL-NA and 61% yield (8.3 mg, 0.01 mmol) for 6SL-NA. Q-TOF-MS *m/z* for 3SL-NA ($C_{33}H_{48}N_2O_{18}$): calcd. $[M+H]^+$ 761.2975, found 761.3001. Analytical HPLC gave T_R of 8.937 min over 12 min with 10%–90% elute B, and purity of 97.7%. Q-TOF-MS *m/z* for 6SL-NA ($C_{33}H_{48}N_2O_{18}$): calcd. $[M+H]^+$ 761.2975, found 761.3022. Analytical HPLC gave T_R of 8.593 min over 12 min with 10%–90% elute B, and purity of 98.3%.

Synthesis and characterization of 3SL-Py and 6SL-Py

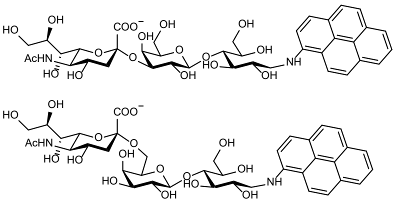

Sialylted trisaccharide (3SL and 6SL) derivatives with the pyrene (Py) tag (*i.e.*, **3SL-Py** and **6SL-Py**) were prepared according to the *general procedure* from 3'-sialyllactose or 6'-sialyllactose (10 mg) with 1-aminopyrene (6.6 mg) in the presence of $NaBH_3CN$ (4.8 mg). The gradient elution condition of HPLC separation is 30%–85% elute B in 12 minutes. The collected fraction was concentrated and subjected to freeze drying to obtain the target product in 62% yield (9.3 mg, 0.011 mmol) for 3SL-Py and 65% yield (9.7 mg, 0.012 mmol) for 6SL-Py. Q-TOF-MS *m/z* for 3SL-Py ($C_{39}H_{50}N_2O_{18}$): calcd. $[M+H]^+$ 835.3131, found 835.3075. Analytical HPLC gave T_R of 10.625 min over 12 min with 10%–90% elute B, and purity of 94.8%. Q-TOF-MS *m/z* for 6SL-Py ($C_{39}H_{50}N_2O_{18}$): calcd. $[M+H]^+$ 835.3131, found 835.3053. Analytical HPLC gave T_R of 8.593 min over 12 min with 10%–90% elute B, and purity of 98.3%.

Synthesis and characterization of 3SL-TPE and 6SL-TPE

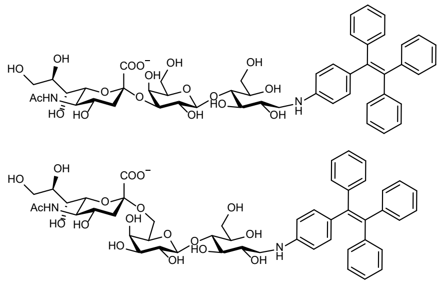

Sialylted trisaccharide (3SL and 6SL) derivatives with the tetraphenylethylene (TPE) tag (*i.e.*, **3SL-TPE** and **6SL-TPE**) were prepared according to the *general procedure* from 3'-sialyllactose or 6'-sialyllactose (10 mg) with 1-(4-aminophenyl)-1,2,2-triphenylethene (10.6 mg) in the presence of $NaBH_3CN$ (4.8 mg). The gradient elution

condition of HPLC separation is 40%–90% (10th min)–90% (13th min) elute B in 13 minutes. The collected fraction was concentrated and subjected to freeze drying to obtain the target product in 75% yield (13 mg, 0.014 mmol) for 3SL-TPE and 69% yield (12 mg, 0.012 mmol) for 6SL-TPE. Q-TOF-MS *m/z* for 3SL-TPE ($C_{49}H_{60}N_2O_{18}$): calcd. 965.3914, found 965.4042. Analytical HPLC gave T_R of 12.314 min over 14 min with 10%–90% elute B, and purity of 97.0%. Q-TOF-MS *m/z* for 6SL-TPE ($C_{49}H_{60}N_2O_{18}$): calcd. 965.3914, found 965.4019. Analytical HPLC gave T_R of 12.070 min over 14 min with 10%–90% elute B, and purity of 99.6%.

Synthesis and characterization of 3SL-NP and 6SL-NP

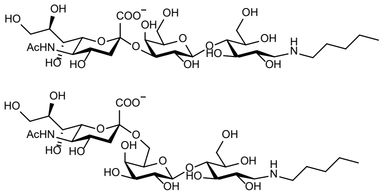

Sialylted trisaccharide (3SL and 6SL) derivatives with the n-pentane (NP) tag (*i.e.*, **3SL-NP** and **6SL-NP**) were prepared according to the *general procedure* from 3'-Sialyllactose or 6'-Sialyllactose (10 mg) with 1-amylamine (2.7 mg) in the presence of $NaBH_3CN$ (4.8 mg). The gradient elution condition of HPLC separation is 10%–48% elute B in 9 minutes. The UV/Vis detection wavelength was set to 195 nm. The collected fraction was concentrated and subjected to freeze drying to obtain the target product in 55% yield (6.9 mg, 0.01 mmol) for 3SL-NP and 60% yield (7.6 mg, 0.01 mmol) for 6SL-NP. Q-TOF-MS *m/z* for 3SL-NP ($C_{28}H_{52}N_2O_{18}$): calcd. $[M-H]^-$ 703.3142, found 703.3152. Analytical HPLC (UV/Vis detection wavelength is 195 nm) gave T_R of 8.816 min over 12 min with 5%–80% elute B, and purity of 98.3%. Q-TOF-MS *m/z* for 6SL-NP ($C_{28}H_{52}N_2O_{18}$): calcd. $[M+H]^+$ 705.3288, found 705.3417. Analytical HPLC (UV/Vis detection wavelength is 195 nm) gave T_R of 8.716 min over 12 min with 5%–80% elute B, and purity of 96.4%.

Synthesis and characterization of 3SG-MPB and 6SG-MPB

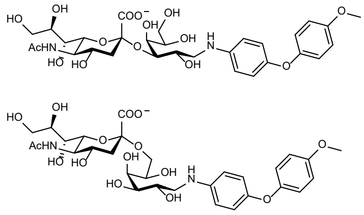

Sialylted disaccharide (3SG and 6SG) derivatives with MPB tag (*i.e.*, **3SG-MPB** and **6SG-MPB**) were prepared according to the *general procedure* from 3'-Sialylgalactose (3SG) or 6'-Sialylgalactose (6SG) (5 mg) with 4-(4-methoxyphenoxy) aniline (4.4 mg) in the presence of $NaBH_3CN$ (3.4 mg). The gradient elution condition of HPLC separation is 20%–74% elute B in 12 minutes. The collected fraction was concentrated and subjected to freeze drying to obtain the target product in 58% yield (3.9 mg, 0.006 mmol) for 3SG-MPB and 53% yield (3.6 mg, 0.005 mmol) for 6SG-MPB. Q-TOF-MS *m/z* for 3SG-MPB ($C_{30}H_{42}N_2O_{15}$): calcd. $[M+H]^+$ 671.2658, found 671.2634. Analytical HPLC gave T_R of 10.236 min over 12 min with 10%–90% elute B, and purity of 97.9%. Q-TOF-MS *m/z* for 6SG-MPB ($C_{30}H_{42}N_2O_{15}$): calcd. $[M+H]^+$ 671.2658, found 671.2626. Analytical HPLC gave T_R of 10.376 min over 12 min with 10%–90% elute B, and purity of 97.6%.

Synthesis and characterization of Lac-MPB

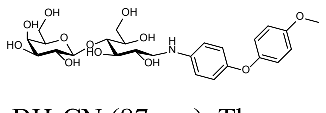

Lactose (Lac) derivative with MPB tag (*i.e.*, **Lac-MPB**) was prepared according to the *general procedure* from D-lactose monohydrate (100 mg) with 4-(4-methoxyphenoxy) aniline (120.0 mg) in the presence of $NaBH_3CN$ (87 mg). The gradient elution condition of HPLC separation is 20%–74% elute B in 12 minutes. The collected fraction was concentrated and subjected to freeze drying to obtain the product Lac-MPB in 65% yield (97 mg, 0.18 mmol). Q-TOF-MS *m/z* ($C_{25}H_{35}NO_{12}$): calcd. $[M+H]^+$ 542.2232, found 542.2393. Analytical HPLC gave T_R of 10.337 min over 12 min with 10%–90% elute B, and purity of 99.8%.

Synthesis and characterization of 2FL-MPB

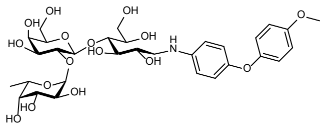

2'-Fucosyllactose (2FL) derivative with MPB tag (*i.e.*, **2FL-MPB**) was prepared according to the *general procedure* from 2FL (10 mg) with 4-(4-methoxyphenoxy) aniline (8.8 mg) in the presence of $NaBH_3CN$ (6.4 mg). The gradient elution condition of HPLC separation is 20%–74% elute B in 12 minutes. The collected fraction was concentrated and subjected to freeze drying to obtain the product 2FL-MPB in 68% yield (9.5 mg, 0.014 mmol). Q-TOF-MS *m/z* ($C_{31}H_{45}NO_{16}$): calcd. $[M+H]^+$ 688.2811, found 688.2784. Analytical HPLC gave T_R of 10.202 min over 12 min with 10%–90% elute B, and purity of 99.6%.

Synthesis and characterization of 3FL-MPB

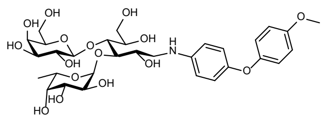

3'-Fucosyllactose (3FL) derivative with MPB tag (*i.e.*, **3FL-MPB**) was prepared according to the *general procedure* from 3FL (10 mg) with 4-(4-methoxyphenoxy) aniline (8.8 mg) in the presence of $NaBH_3CN$ (6.4 mg). The gradient elution condition of HPLC separation is 20%–70% elute B in 12 minutes. The collected fraction was concentrated and subjected to freeze drying to obtain the product 3FL-MPB in 74% yield (10.4 mg, 0.015 mmol). Q-TOF-MS *m/z* ($C_{31}H_{45}NO_{16}$): calcd. $[M+H]^+$ 688.2811, found 688.2872. Analytical HPLC gave T_R of 10.057 min over 12 min with 10%–90% elute B, and purity of 99.7%.

Synthesis and characterization of STetra2-MPB

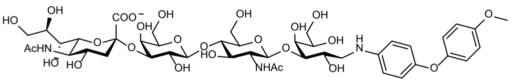

STetra2 derivative with MPB tag (*i.e.*, **STetra2-MPB**) was prepared according to the *general procedure* from STetra2 (3 mg) with 4-(4-methoxyphenoxy) aniline (1.5 mg) in the presence of $NaBH_3CN$ (1.1 mg). The gradient elution condition of HPLC separation is 26%–72% elute B in 10 minutes. The collected fraction was concentrated and subjected to freeze drying to obtain the product STetra2-

MPB in 67% yield (2.4 mg, 0.002 mmol). Q-TOF-MS *m/z* ($C_{44}H_{65}N_3O_{25}$): calcd. $[M+H]^+$ 1036.3987, found 1036.3977. Analytical HPLC gave T_R of 9.936 min over 12 min with 10%–90% elute B, and purity of 99.7%.

Synthesis and characterization of LSTa-MPB

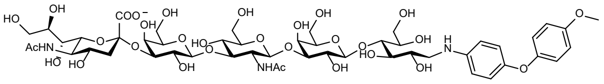

LSTa derivative with MPB tag (*i.e.*, **LSTa-MPB**) was prepared according to the *general procedure* from LSTa (3 mg) with 4-(4-methoxyphenoxy) aniline (1.3 mg) in the presence of $NaBH_3CN$ (1 mg). The gradient elution condition of HPLC separation is 26%–72% elute B in 10 minutes. The collected fraction was concentrated and subjected to freeze drying to obtain the product LSTa-MPB in 67% yield (2.1 mg, 0.0017 mmol). Q-TOF-MS *m/z* ($C_{50}H_{75}N_3O_{30}$): calcd. $[M+H]^+$ 1198.4508, found 1198.4535. Analytical HPLC gave T_R of 9.959 min over 12 min with 10%–90% elute B, and purity of 99.8%.

Synthesis and characterization of LSTb-MPB

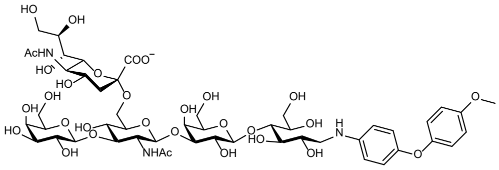

LSTb derivative with MPB tag (*i.e.*, **LSTb-MPB**) was prepared according to the *general procedure* from LSTb (1 mg) with 4-(4-methoxyphenoxy) aniline (0.5 mg) in the presence of $NaBH_3CN$ (0.5 mg). The gradient elution condition of HPLC separation with a BOSTON Green ODS column (4.6 mm × 250 mm, 10 μm, 1 mL·min^{-1}) is 20%–80% elute B in 12 minutes. The collected fraction was concentrated and subjected to freeze drying to obtain the product LSTb-MPB. Q-TOF-MS *m/z* ($C_{50}H_{75}N_3O_{30}$): calcd. $[M+H]^+$ 1198.4508, found 1198.4501. Analytical HPLC gave T_R of 7.262 min over 12 min with 10%–90% elute B, and purity of 99.4%.

Synthesis and characterization of LSTc-MPB

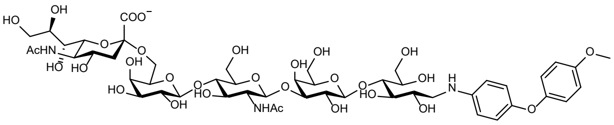

LSTc derivative with MPB tag (*i.e.*, **LSTc-MPB**) was prepared according to the *general procedure* from LSTc (1 mg) with 4-(4-methoxyphenoxy) aniline (0.5 mg) in the presence of $NaBH_3CN$ (0.5 mg). The gradient elution condition of HPLC separation with a BOSTON Green ODS column (4.6 mm × 250 mm, 10 μm, 1 mL·min^{-1}) is 20%–80% elute B in 12 minutes. The collected fraction was concentrated and subjected to freeze drying to obtain the product LSTc-MPB. Q-TOF-MS *m/z* ($C_{50}H_{75}N_3O_{30}$): calcd. $[M+H]^+$ 1198.4508, found 1198.4511. Analytical HPLC gave T_R of 7.293 min over 12 min with 10%–90% elute B, and purity of 99.2%.

Synthesis and characterization of LSTd-MPB

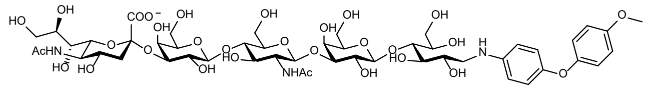

LSTd derivative with MPB tag (*i.e.*, **LSTd-MPB**) was prepared according to the *general procedure* from LSTd (1 mg) with 4-(4-methoxyphenoxy) aniline (0.5 mg) in the presence of $NaBH_3CN$ (0.5 mg). The gradient elution condition of HPLC separation with a BOSTON Green ODS column (4.6 mm × 250 mm, 10 μm, 1 mL·min^{-1}) is 20%–80% elute B in 12 minutes. The collected fraction was concentrated and subjected to freeze drying to obtain the product LSTd-MPB. Q-TOF-MS *m/z* ($C_{50}H_{75}N_3O_{30}$): calcd. $[M+H]^+$ 1198.4508, found 1198.4533. Analytical HPLC gave T_R of 7.332 min over 12 min with 10%–90% elute B, and purity of 98.9%.

Synthesis and characterization of 6SL-DPE-NH_2

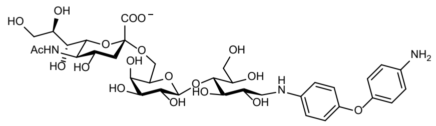

6SL derivative with the 4-aminodiphenyl ether label (*i.e.*, **6SL-DPE-NH_2**) was prepared according to the *general procedure* and some minor modification from 6SL (100 mg) and 4,4'-diaminodiphenyl ether (DADPE) (305 mg) in the presence of $NaBH_3CN$ (50 mg). Here, 10 equiv. DADPE was added into the reaction solution to ensure the reaction of 6SL with only one amino group. The gradient elution condition of HPLC separation is 10%–40% elute B in 10 minutes. The collected fraction was concentrated and subjected to freeze drying to obtain the product 6SL-DPE-NH_2 in 56% yield (82 mg, 0.1 mmol). Q-TOF-MS *m/z* ($C_{35}H_{51}N_3O_{19}$): calcd. $[M+H]^+$ 818.3190, found 818.3212. Analytical HPLC gave T_R of 8.035 min over 12 min with 5%–90% elute B, and purity of 90.6%.

Synthesis and characterization of Lac-DPE-6SL

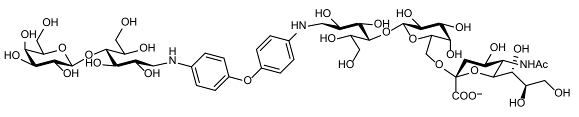

Lactose (Lac) derivative with a composite tag from 6SL-DPE-NH_2 was prepared according to the *general procedure* from D-lactose monohydrate (3 mg) and 6SL-DPE-NH_2 (10mg) in the presence of $NaBH_3CN$ (2.8 mg). Here, 6SL-DPE-NH_2 with an amino group was used as the labelling regent. The resulting lactose derivative was denoted as **Lac-DPE-6SL**. The gradient elution condition of HPLC separation is 5%–35% elute B in 10 minutes. The collected fraction was concentrated and subjected to freeze drying to obtain the product Lac-DPE-6SL in 62% yield (5.9 mg, 0.005 mmol). Q-TOF-MS *m/z* ($C_{47}H_{73}N_3O_{29}$): calcd. $[M+H]^+$ 1144.4402, found 1144.4292. Analytical HPLC gave T_R of 14.576 min over 12 min with 10%–90% elute B, and purity of 99.3%.

Synthesis and characterization of Cel-DPE-6SL

Cellobiose (Cel) derivative with a composite tag from 6SL-DPE-NH_2 was prepared according to the *general procedure* from D-(+)-cellobiose (3 mg) and 6SL-DPE-NH_2 (10 mg) in the presence of $NaBH_3CN$ (2.8 mg). The resulting cellobiose derivative was denoted as **Cel-DPE-6SL**. The gradient elution condition of HPLC separation is 5%–35% elute B in 10 minutes. The collected fraction was concentrated and subjected to freeze drying to obtain the product Cel-DPE-6SL in 66% yield (6.7 mg, 0.0057 mmol). Q-TOF-MS *m/z* ($C_{47}H_{73}N_3O_{29}$): calcd. $[M+H]^+$ 1144.4402, found 1144.4334. Analytical HPLC gave T_R of 4.449 min over 12 min with 10%–90% elute B, and purity of 98.7%.

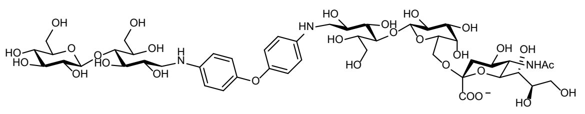

Synthesis and characterization of Mal-DPE-6SL

Maltose (Mal) derivative with a composite tag from 6SL-DPE-NH_2 was prepared according to the *general procedure* from maltose monohydrate (3 mg) and 6SL-DPE-NH_2 (10 mg) in the presence of $NaBH_3CN$ (2.8 mg). The resulting maltose derivative was denoted as **Mal-DPE-6SL**.The gradient elution condition of HPLC separation is 5%–35% elute B in 10 minutes. The collected fraction was concentrated and subjected to freeze drying to obtain the product Mal-DPE-6SL in 54% yield (5.1 mg, 0.0045 mmol). Q-TOF-MS *m/z* ($C_{47}H_{73}N_3O_{29}$): calcd. $[M+H]^+$ 1144.4402, found 1144.4367. Analytical HPLC gave T_R of 4.404 min over 12 min with 10%–90% elute B, and purity of 99.6%.

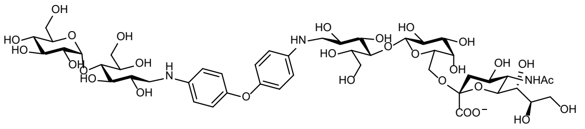

Synthesis and characterization of LeA-DPE-6SL

Lewis A (LeA) triaose derivative with a composite tag from 6SL-DPE-NH_2 was prepared according to the *general procedure* from LeA (1 mg) and 6SL-DPE-NH_2 (2 mg) in the presence of $NaBH_3CN$ (1 mg). The resulting LeA derivative was denoted as **LeA-DPE-6SL**.The gradient elution condition of HPLC separation is 5%–35% elute B in 11 minutes. The collected fraction was concentrated and subjected to freeze drying to obtain the product. Q-TOF-MS *m/z* ($C_{55}H_{86}N_4O_{33}$): calcd. $[M+H]^+$ 1331.5247, $[M+2H]^{2+}$ 666.2660, found $[M+H]^+$ 1331.5252, $[M+2H]^{2+}$ 666.2671. Analytical HPLC gave T_R of 8.937 min over 15 min with 5%–80% elute B, and purity of 98.5%.

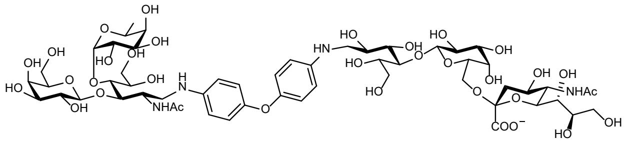

Synthesis and characterization of LeX-DPE-6SL

Lewis X (LeX) triaose derivative with a composite tag from 6SL-DPE-NH_2 was

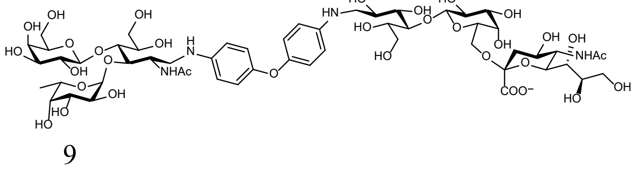

prepared according to the *general procedure* from LeX (1 mg) and 6SL-DPE-NH_2 (2 mg) in the presence of $NaBH_3CN$ (1 mg). The resulting LeX derivative was denoted as **LeX-DPE-6SL**. The gradient elution condition of HPLC separation is 5%–35% elute B in 11 minutes. The collected fraction was concentrated and subjected to freeze drying to obtain the product. Q-TOF-MS *m/z* ($C_{55}H_{86}N_4O_{33}$): calcd. $[M+H]^+$ 1331.5247, $[M+2H]^{2+}$ 666.2660, found $[M+H]^+$ 1331.5243, $[M+2H]^{2+}$ 645.2670. Analytical HPLC gave T_R of 6.157 min over 15 min with 5%–80% elute B, and purity of 97.8%.

Synthesis and characterization of LNnT-DPE-6SL

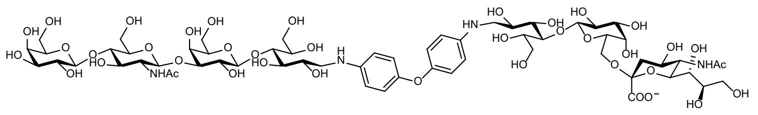

Lacto-N-neotetraose (LNnT) derivative with a composite tag from 6SL-DPE-NH_2 was prepared according to the *general procedure* from LNnT (5 mg) and 6SL-DPE-NH_2 (8 mg) in the presence of $NaBH_3CN$ (2.8 mg). The resulting LNnT derivative was denoted as **LNnT-DPE-6SL**.The gradient elution condition of HPLC separation is 5%–35% elute B in 10 minutes. The collected fraction was concentrated and subjected to freeze drying to obtain the product LNnT-DPE-6SL in 65% yield (6.9 mg, 0.0046 mmol). Q-TOF-MS *m/z* ($C_{61}H_{96}N_4O_{39}$): calcd. $[M+2H]^{2+}$ 755.2899, found $[M+2H]^{2+}$ 755.2935. Analytical HPLC gave T_R of 4.368 min over 12 min with 10%–90% elute B, and purity of 98.4%.

Synthesis and characterization of LNT-DPE-6SL

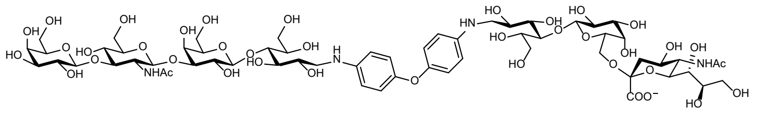

Lacto-N-tetraose (LNT) derivative with a composite tag from 6SL-DPE-NH_2 was prepared according to the *general procedure* from LNT (2 mg) and 6SL-DPE-NH_2 (3 mg) in the presence of $NaBH_3CN$ (1 mg). The resulting LNT derivative was denoted as **LNT-DPE-6SL**.The gradient elution condition of HPLC separation with a BOSTON Green ODS column (4.6 mm × 250 mm, 10 μm, 1 mL·min^{-1}) is 5%–43% elute B in 10 minutes. The collected fraction was concentrated and subjected to freeze drying to obtain the product LNT-DPE-6SL. Q-TOF-MS *m/z* ($C_{61}H_{96}N_4O_{39}$): calcd. $[M+2H]^{2+}$ 755.2899, $[M+1H]^+$ 1509.5724, found $[M+2H]^{2+}$ 755.2909, $[M+1H]^+$ 1509.5710. Analytical HPLC gave T_R of 7.919 min over 12 min with 5%–80% elute B, and purity of 97.9Z%.

Enzymatic synthesis and characterization of 6SL-MPB

α2–6-Sialylation reaction of **Lac-MPB** with Pd26ST was performed to generate **6SL-MPB**. The generation was confirmed by the MS characterization. Q-TOF-MS *m/z* ($C_{36}H_{52}N_2O_{20}$): calcd. for $[M+H]^+$ 833.3186, found 833.3250.

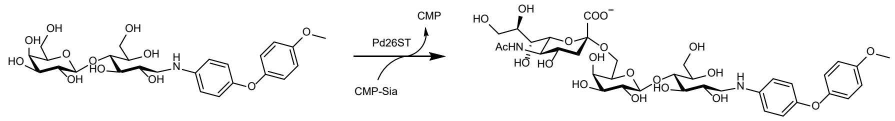

Enzymatic synthesis and characterization of 3SL-MPB

α2–3-Sialylation reaction of **Lac-MPB** with PmST1 was performed to generate **3SL-MPB**. The generation was confirmed by the MS characterization. Q-TOF-MS *m/z* ($C_{36}H_{52}N_2O_{20}$): calcd. for $[M+H]^+$ 833.3186, found 833.3116.

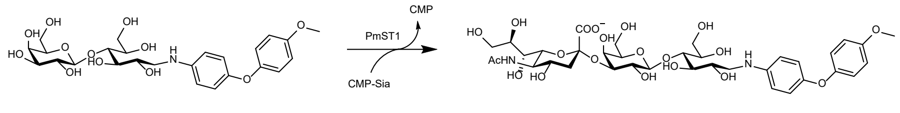

Enzymatic synthesis and characterization of 6S2FL-MPB

Neu5Acα2–6(Fucα1–2)Galβ1–4Glc-MPB (abbreviated as **6S2FL-MPB**) was synthesized through a α2–6-sialylation reaction of **2FL-MPB** with Pd26ST. The generation was confirmed by the MS characterization. Q-TOF-MS *m/z* ($C_{42}H_{62}N_2O_{24}$): calcd. for $[M+H]^+$ 979.3765, found 979.3748.

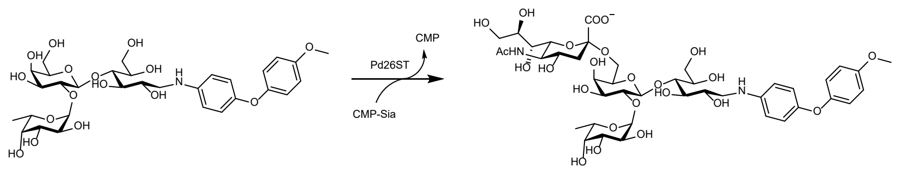

Enzymatic synthesis and characterization of 6S3FL-MPB

Neu5Acα2–6Galβ1–4(Fucα1–3)Glc-MPB (abbreviated as **6S3FL-MPB**) was synthesized through a α2–6-sialylation reaction of **3FL-MPB** with Pd26ST. The generation was confirmed by the MS characterization. Q-TOF-MS *m/z* ($C_{42}H_{62}N_2O_{24}$): calcd. for $[M+H]^+$ 979.3765, found 979.3772.

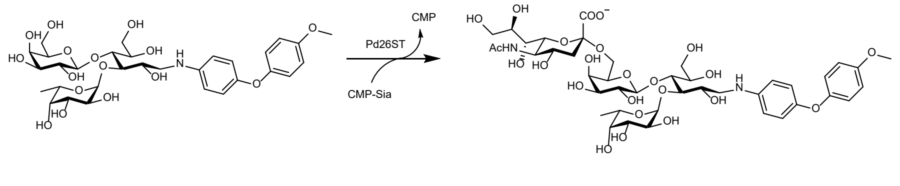

Enzymatic synthesis and characterization of 3S3FL-MPB

Neu5Acα2–3Galβ1–4(Fucα1–3)Glc-MPB (abbreviated as **3S3FL-MPB**) was synthesized through a α2–3-sialylation reaction of **3FL-MPB** with PmST1. The generation was confirmed by the MS characterization. Q-TOF-MS *m/z* ($C_{42}H_{62}N_2O_{24}$): calcd. for $[M+H]^+$ 979.3765, found 979.3745.

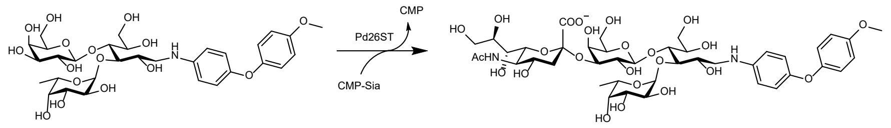

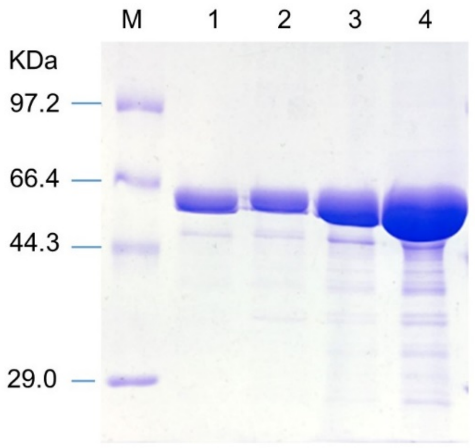

Supplementary Fig. 1. SDS PAGE analysis of recombinant WT proaerolysin. M: marker, 1, 2, 3, and 4 are samples from four collection tubes during separation. This experiment was repeated independently at least three times.

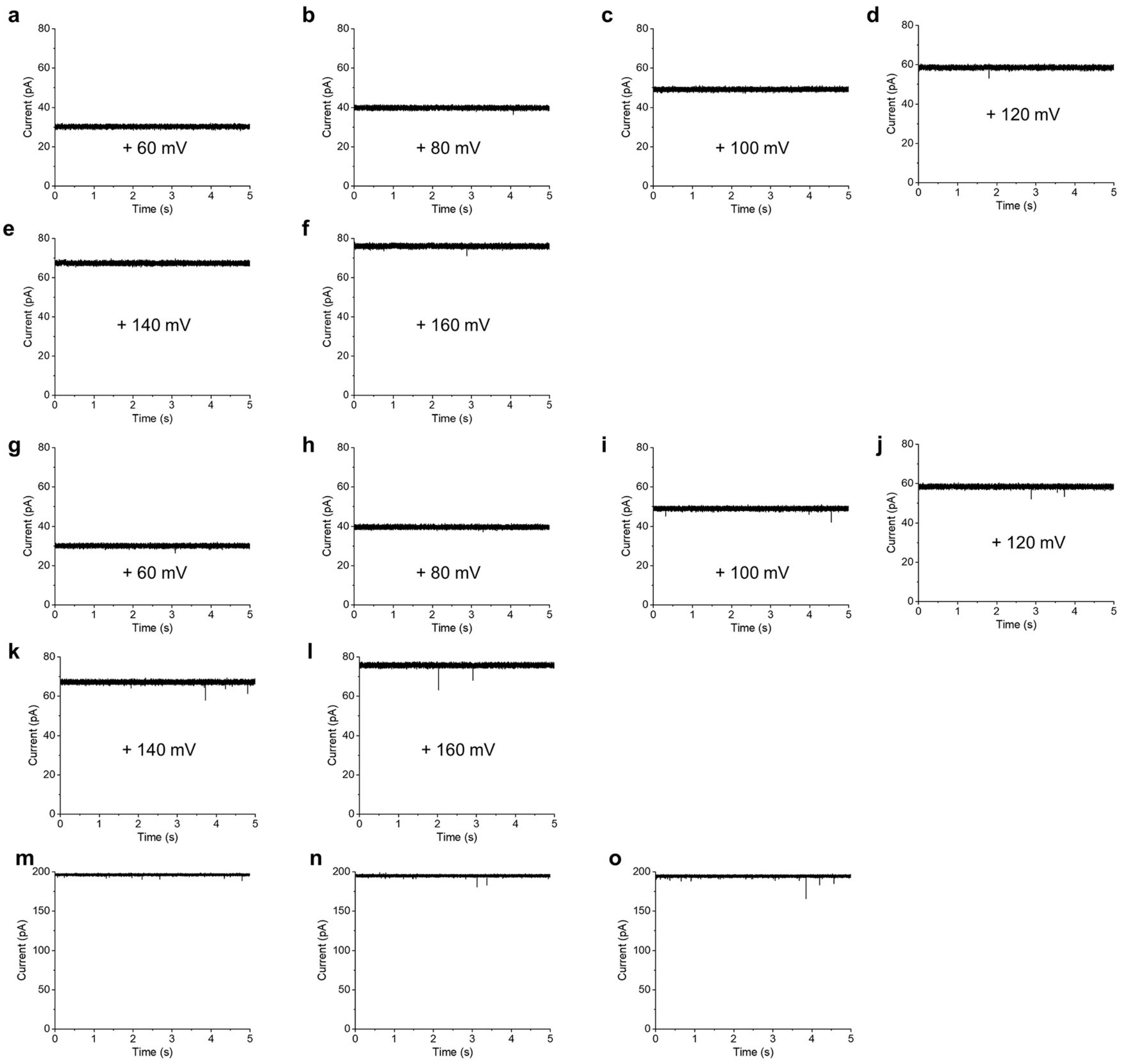

Supplementary Fig. 2. Representative current traces of WT AeL under varying voltages or different salt concentrations. **a-l**, Representative current traces under varied voltages upon addition of 5 μM 3SL (**a-f**) or 5 μM 6SL (**g-l**) into 1 M KCl electrolyte solution (10 mM Tris-HCl buffer containing 1 mM EDTA and 1 M KCl with pH 8.0). **m-o**, Representative current traces in 4 M KCl electrolyte solution without (**m**) and with 5 μM 3SL (**n**) or 5 μM 6SL (**o**) at +100 mV. Here, 4 M KCl electrolyte is a saturated KCl solution prepared in 10 mM Tris-HCl containing 1 mM EDTA and 4 M KCl with pH 8.0. All nanopore data were recorded using a 250kHz sampling rate with a 5 kHz low-pass filtering.

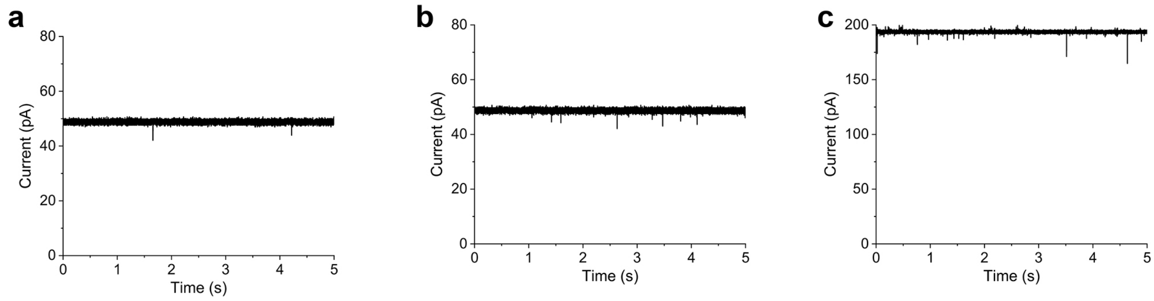

Supplementary Fig. 3. Representative current traces of WT AeL upon addition of analytes with different concentration or different salt concentrations. Representative current traces upon addition of 2 µM LSTa (**a**) or 10 µM LSTa (**b**) in the electrolyte solution containing 1M KCl (10 mM Tris-HCl buffer containing 1 mM EDTA and 1 M KCl with pH 8.0), or 10 µM LSTa in electrolyte containing 4 M KCl (**c**), at +100 mV. All nanopore data were recorded using a 250kHz sampling rate with a 5 kHz low-pass filtering.

Supplementary Note 1

We found in the reported literatures that higher applied voltage, higher analyte concentration, or higher salt concentration can significantly increase the current blockage frequency. For example, a report from Prof. Juan Pelta's group showed that the events frequency of dextran sulfate transported through AeL nanopore increased exponentially as a function of applied voltage and linearly as a function of dextran sulfate concentration[1]. A work from Prof. Xiyun Guan's group reported the increase of in the event frequency of a peptide traversing hemolysin nanopore was observed when the concentration of NaCl electrolyte solution from 1M to 3M[2]. Thus, we attempted to increase the applied voltage (from +60 mV to +160 mV) (Supplementary Fig. 2), the salt concentration (from 1M KCl to 4M KCl) (Supplementary Fig. 3), and the analyte concentration (from 2 μM to 10 μM) to explore whether the obvious blockage signals could be observed. After these, we still cannot observe the obvious blockage signals from 3SL, 6SL, or LSTa, by combining the results of Supplementary Figs. 2 and 3.

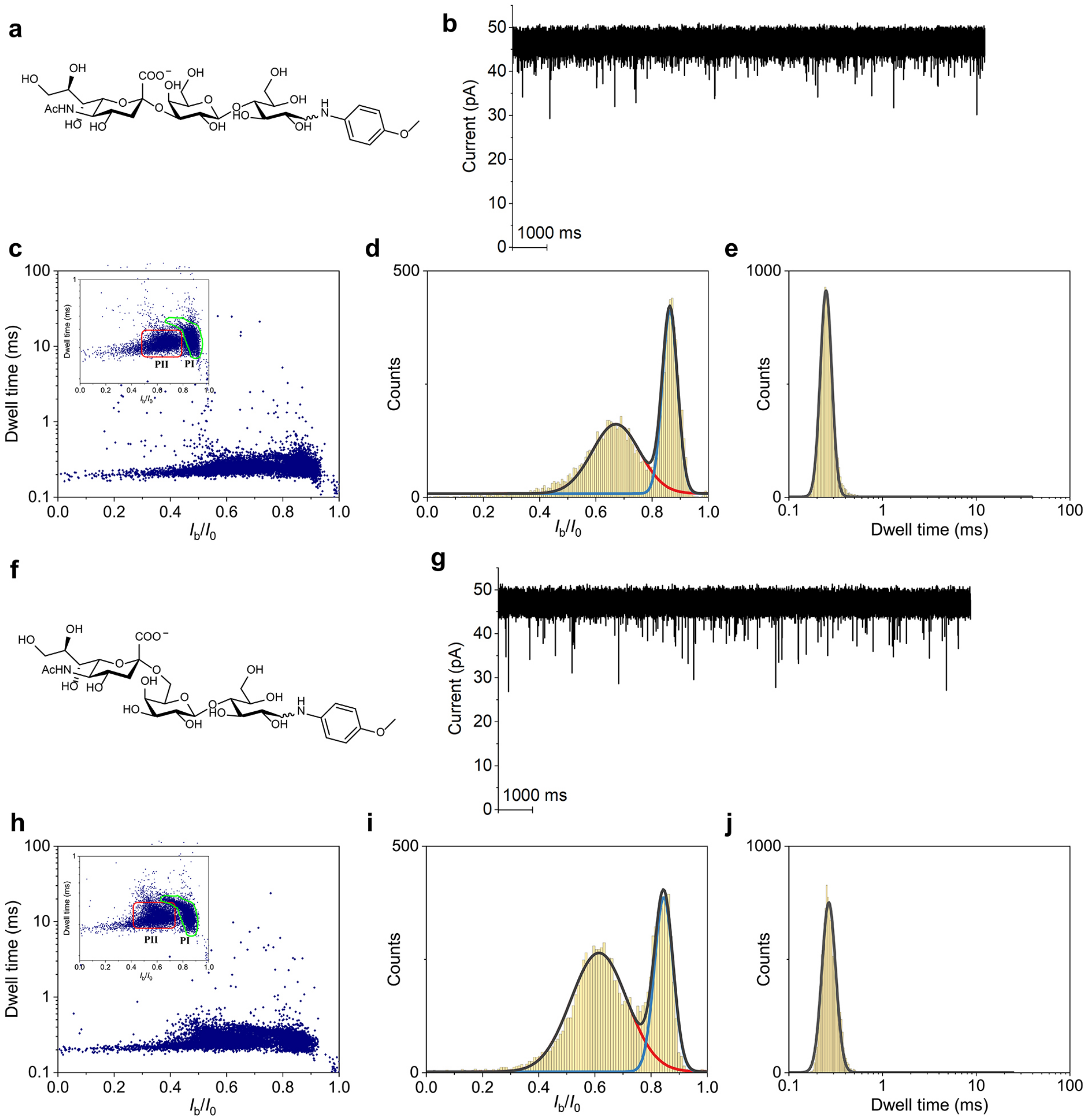

Supplementary Fig. 4. AeL nanopore test results of 3SL-MB and 6SL-MB. **a-e,** Chemical structure of 3SL-MB (**a**), and the representative nanopore ionic current traces (**b**), scatter plot of I_b/I_0 *vs.* dwell time (**c**), and the corresponding distribution of I_b/I_0 (**d**) and dwell time (**e**) from nanopore measurement of 3SL-MB. **f-j**, Chemical structure of 6SL-MB (**f**), and the representative nanopore ionic current traces (**g**), scatter plot of I_b/I_0 *vs.* dwell time (**h**), and distribution of I_b/I_0 (**i**) and dwell time (**j**) from nanopore measurement of 6SL-MB. Insets of **c** and **h** are the enlarged scatter plots. Measurements were done in a 10 mM Tris-HCl buffer containing 1 mM EDTA and 1 M KCl with pH 8.0 at +100 mV voltage. All nanopore data were recorded using a 250kHz sampling rate with a 5 kHz low-pass filtering. Each scatter plot contains at least 9,000 events.

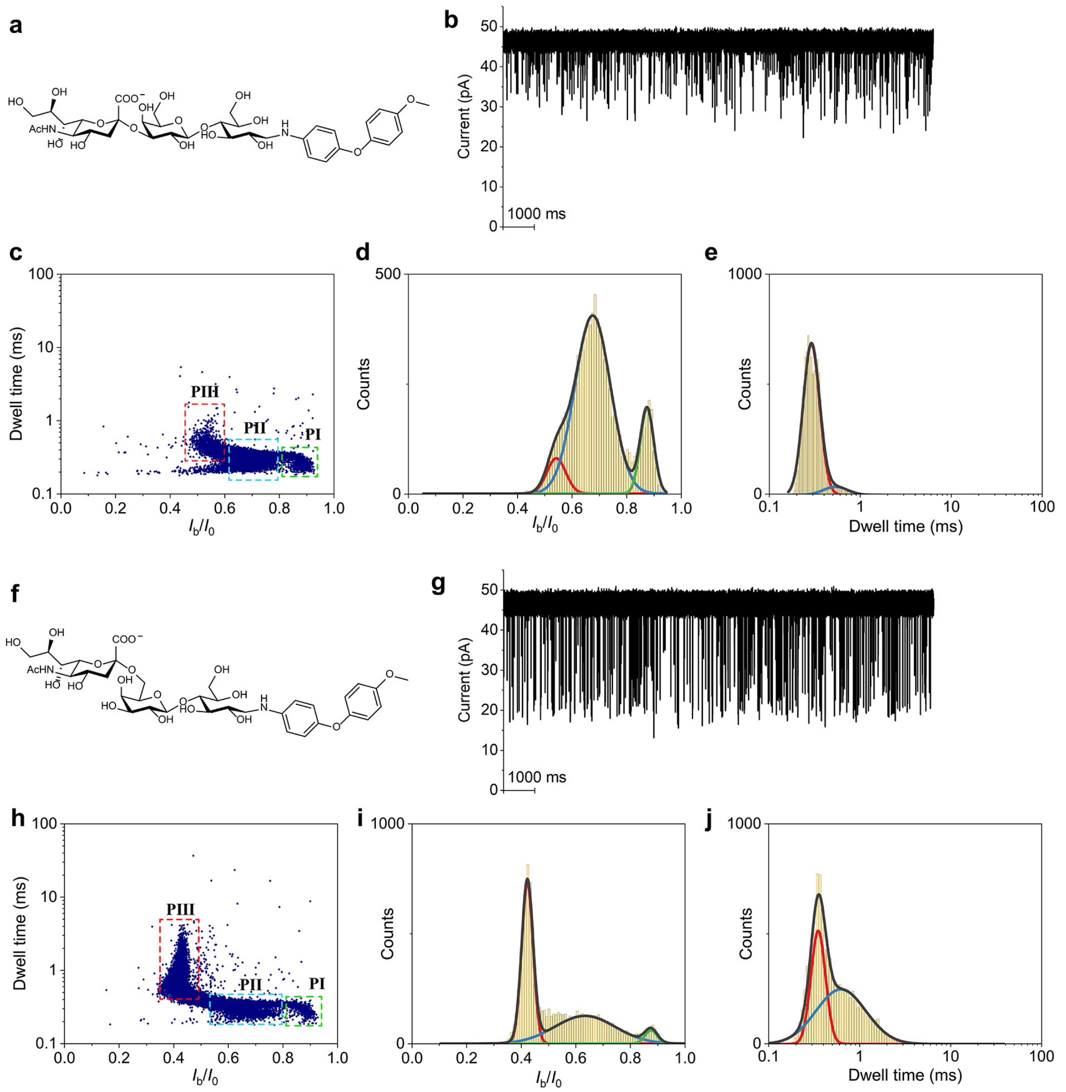

Supplementary Fig. 5. AeL nanopore test results of 3SL-MPB and 6SL-MPB. **a-e,** Chemical structure of 3SL-MPB (**a**), and the representative current traces (**b**), scatter plot of I_b/I_0 *vs.* dwell time (**c**), and the corresponding distribution of I_b/I_0 (**d**) and dwell time (**e**) from nanopore measurement of 3SL-MPB. **f-j,** Chemical structure of 6SL-MPB (**f**), and the representative current traces (**g**), scatter plot of I_b/I_0 *vs.* dwell time (**h**), and distribution of I_b/I_0 (**i**) and dwell time (**j**) from nanopore measurement of 6SL-MPB. Measurements were done in a 10 mM Tris-HCl buffer containing 1 mM EDTA and 1 M KCl with pH 8.0 at +100 mV voltage. All nanopore data were recorded using a 250kHz sampling rate with a 5 kHz low-pass filtering. Each scatter plot contains at least 9,000 events.

Supplementary Note 2

Both scatter plots from 3SL-MB and 6SL-MB (Supplementary Fig. 4) show two populations, which are defined as PI and PII. And the I_b/I_0 obeys normal distribution and was fitted to two Gaussian peaks. Among which, PI corresponds to the small blockage current amplitude (large I_b/I_0 value). PII corresponds to the large blockage current amplitude (small I_b/I_0 value). We found that the I_b/I_0 value of PI from 3SL-MB is basically identical to that from 6SL-MB, however, the I_b/I_0 value of PII varied by glycan. The dwell time follows the lognormal distribution, and the corresponding logarithmic dwell time was fitted to single Gaussian peak. Both PI and PII were fitted to a single peak, and the corresponding mean dwell time did not vary by glycan. The I_b/I_0 and dwell time of all other data throughout this work also follow the similar distribution (normal distribution for I_b/I_0, lognormal distribution for dwell time), which will not be repeated elsewhere.

In the scatter plots of 3SL-MPB and 6SL-MPB (Supplementary Fig. 5), aside from PI and PII populations that are similar to that of 3SL-MB or 6SL-MB, there is the third population, PIII, showcasing the obvious difference between 3SL-MPB and 6SL-MPB. Thus, PIII domain can serve as the characteristic population to identify the glycan. The corresponding mean I_b/I_0 value and dwell time can be used to index the glycan.

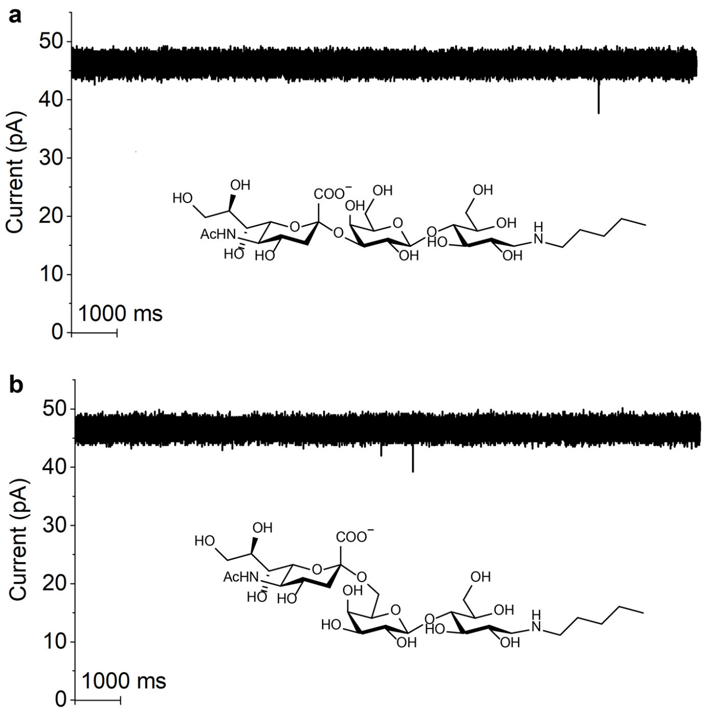

Supplementary Fig. 6. The representative current traces of AeL nanopore towards the measurement of 3SL-NP (**a**) and 6SL-NP (**b**). Measurements were done in a 10 mM Tris-HCl buffer containing 1 mM EDTA and 1 M KCl with pH 8.0 at +100 mV voltage. All nanopore data were recorded using a 250kHz sampling rate with a 5 kHz low-pass filtering.

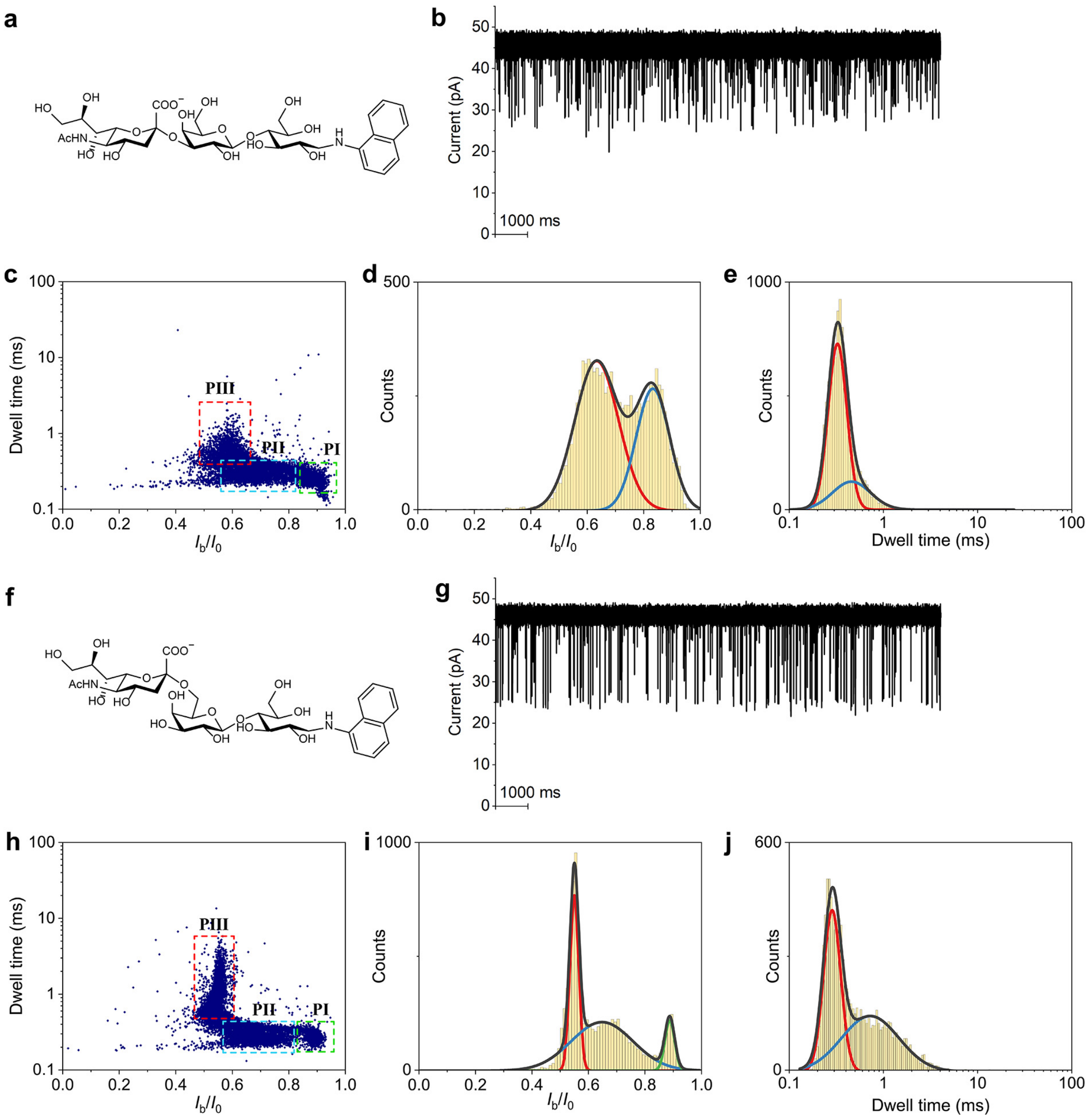

Supplementary Fig. 7. AeL nanopore test results of 3SL-NA and 6SL-NA. **a-e**, Chemical structure of 3SL-NA (**a**), and the representative current traces (**b**), scatter plot of I_b/I_0 *vs.* dwell time (**c**), and the corresponding distribution of I_b/I_0 (**d**) and dwell time (**e**) from nanopore measurement of 3SL-NA. **f-j**, Chemical structure of 6SL-NA (**f**), and the representative current traces (**g**), scatter plot of I_b/I_0 *vs.* dwell time (**h**), and distribution of I_b/I_0 (**i**) and dwell time (**j**) from nanopore measurement of 6SL-NA. Measurements were done in a 10 mM Tris-HCl buffer containing 1 mM EDTA and 1 M KCl with pH 8.0 at +100 mV. All nanopore data were recorded using a 250kHz sampling rate with a 5 kHz low-pass filtering. Each scatter plot contains at least 9,000 events.

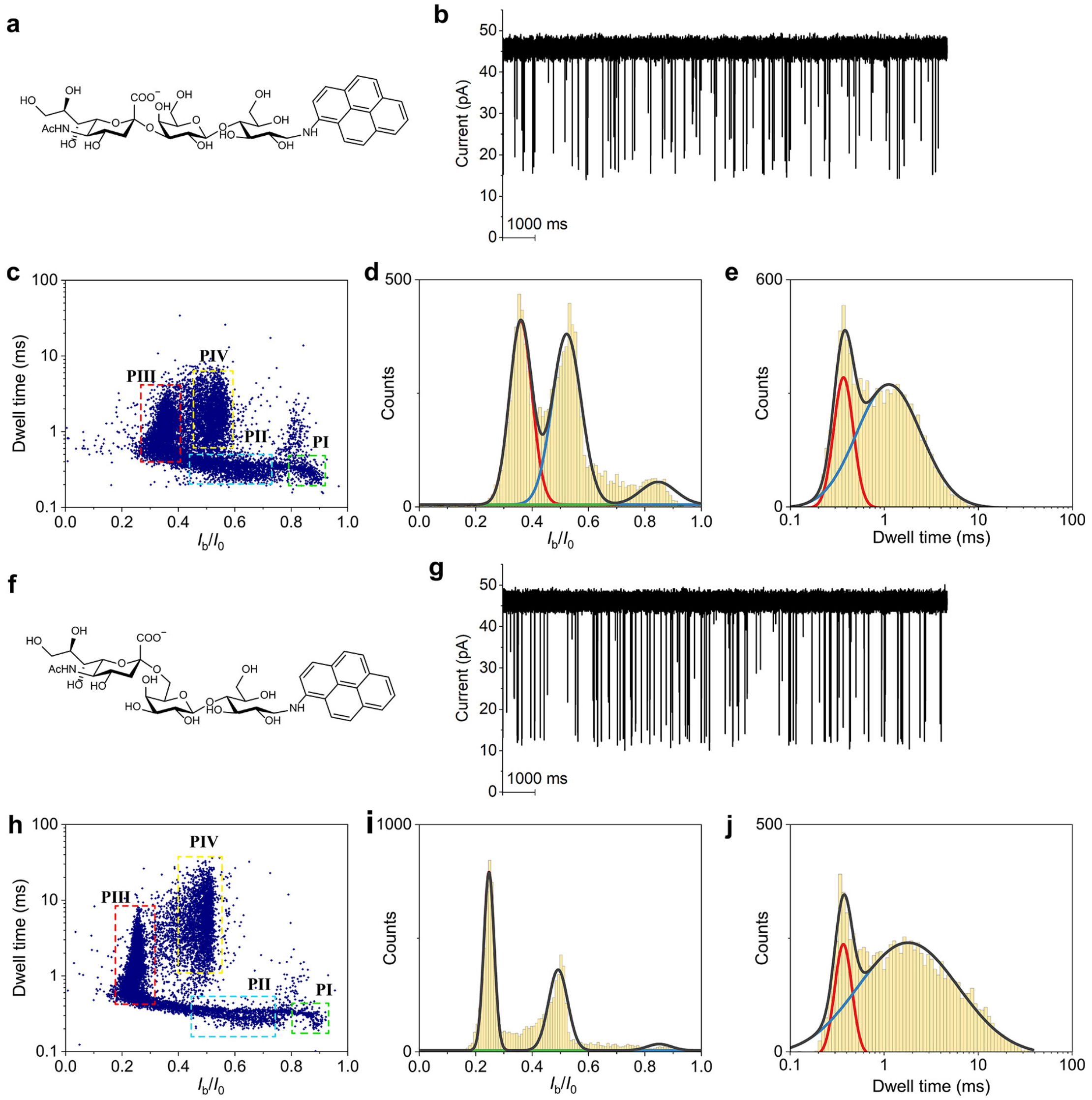

Supplementary Fig. 8. AeL nanopore test results of 3SL-Py and 6SL-Py. **a**-**e**, Chemical structure of 3SL-Py (**a**), and the representative current traces (**b**), scatter plot of I_b/I_0 *vs.* dwell time (**c**), and the corresponding distribution of I_b/I_0 (**d**) and dwell time (**e**) from the nanopore measurement of 3SL-Py. **f**-**j**, Chemical structure of 6SL-Py (**f**), and the representative current traces (**g**), scatter plot of I_b/I_0 *vs.* dwell time (**h**), and distribution of I_b/I_0 (**i**) and dwell time (**j**) from nanopore measurement of 6SL-Py. Measurements were done in a 10 mM Tris-HCl buffer containing 1 mM EDTA and 1 M KCl with pH 8.0 at +100 mV. All nanopore data were recorded using a 250kHz sampling rate with a 5 kHz low-pass filtering. Each scatter plot contains at least 9,000 events.

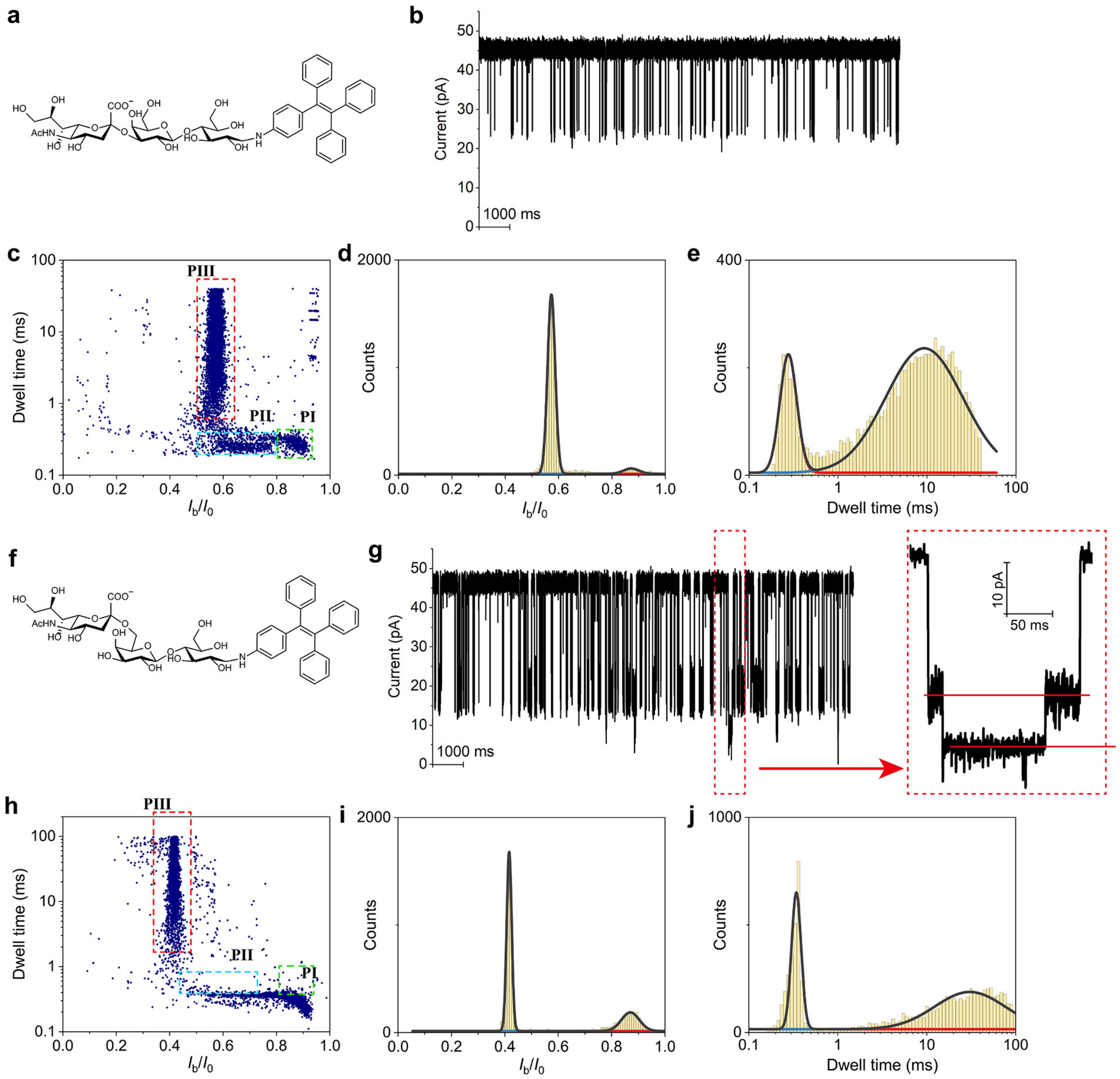

Supplementary Fig. 9. AeL nanopore test results of 3SL-TPE and 6SL-TPE. **a-e**, Chemical structure of 3SL-TPE (**a**), and the representative current traces (**b**), scatter plot of I_b/I_0 *vs.* dwell time (**c**), and the corresponding distribution of I_b/I_0 (**d**) and dwell time (**e**) from the nanopore measurement of 3SL-TPE. **f-j**, Chemical structure of 6SL-TPE (**f**), and the representative current traces (**g**), scatter plot of I_b/I_0 *vs.* dwell time (**h**), and distribution of I_b/I_0 (**i**) and dwell time (**j**) from the nanopore measurement of 6SL-TPE. Inset of (**g**) is an enlarged event with two levels. Measurements were done in a 10 mM Tris-HCl buffer containing 1 mM EDTA and 1 M KCl with pH 8.0 at +100 mV. All nanopore data were recorded using a 100kHz sampling rate with a 5 kHz low-pass filtering. Each scatter plot contains at least 9,000 events.

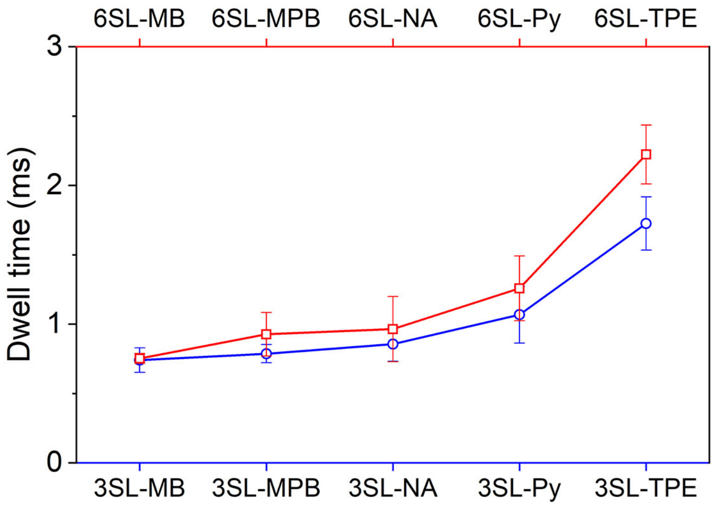

Supplementary Fig. 10. Mean dwell time of 3SL and 6SL with different tags obtained from AeL nanopore measurements. Error bars represents SDs of three independent experiments. The mean dwell times derive from the key and characteristic population–PIII domain–in scatter plot that can show the remarkable difference among glycans. The only exceptions are the MB-tagged glycans, which only exhibited two populations in scatter plots (Supplementary Fig. 4).

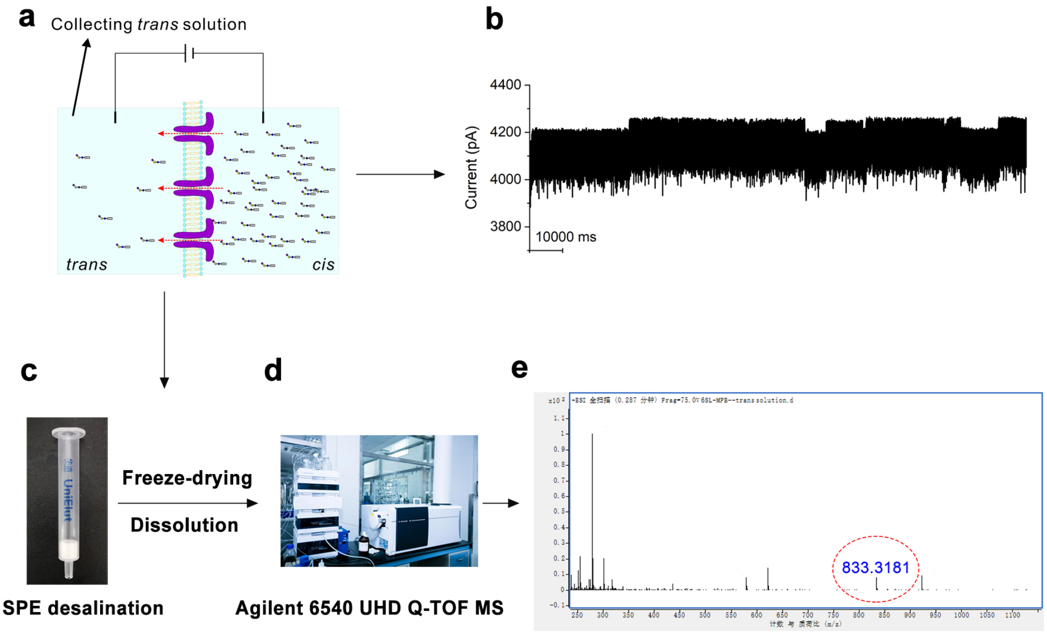

Supplementary Fig. 11. Translocation verification experiment. **a**, Schematic of the translocation experiment through 80~90 AeL nanopores in lipid bilayer membrane. **b**, The representative current traces of many AeL nanopores with the translocation of 6SL-MPB at +100 mV. **c**, Graphic showing the C18-packed solid phase extraction (SPE) micro column. **d**, Graphic showing the used high resolution mass spectrometer: Agilent 6540 UHD quadrupole time-of-flight accurate-mass mass spectrometer. **e**, Partial mass spectrum of the concentrated sample.

Supplementary Note 3

In the translocation verification experiment, 80 ~ 90 AeL nanopores (from the estimation according to the open current) were inserted into the lipid bilayer membrane. Then, 50 µL 2 mM 6SL-MPB solution was added into the electrolyte solution of the *cis* compartment, the final concentration was approximately 100 µM. Under the applied volage of +100 mV, the glycan molecule translocated continually through those AeL nanopores. The recorded current traces are shown in Supplementary Fig. 11b. The experiment lasted about 8.5 hours at +100 mV (During this period, small amount of electrolyte solution was added to the *cis* and *trans* compartments to compensate the volatilized liquid). After that, the electrolyte solution in the *trans* compartment was collected. The collected solution was desalted with C18 SPE micro column (ACCHROM, UniElut C18, 200mg/3mL). Prior to use, the SPE column was washed with 3 mL 85% CH_3CN (v/v)/0.1%(v/v) TFA for activation. Then, 6 mL H_2O/0.1%(v/v) TFA was used for the equilibration of the column. Then, after loading the sample into column, 6 mL H_2O/0.1%(v/v) TFA was used to wash the column to remove the salt. Finally, 3 mL 85% CH_3CN (v/v)/0.1%(v/v) TFA was used to elute the sample. After freeze-drying, the purified sample was dissolved in only 20 µL water to perform MS. The found *m*/*z* 833.3181 verifies the translocation of 6SL-MPB (calcd. for $[M+H]^+$ 833.3186) through AeL nanopore (Supplementary Fig. 11e).

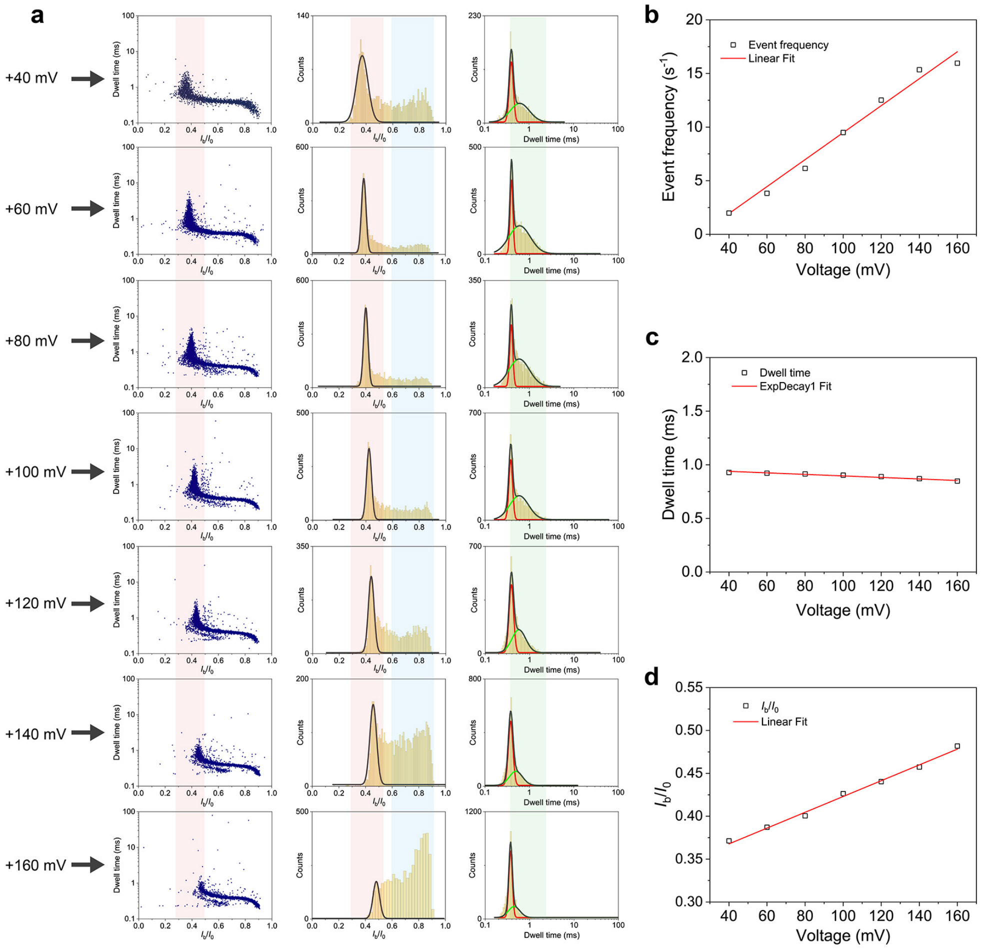

Supplementary Fig. 12. AeL nanopore test results of 6SL-MPB under different voltages. **a**, The scatter plots (left), the histograms of I_b/I_0 (middle) and dwell times (right) for 6SL-MPB at varying potentials ranged from +40 mV to +160 mV. **b-d**, The potential-dependent change of the corresponding event frequency from all blockages (**b**), the dwell time (**c**) and the I_b/I_0 value (**d**) from the PIII domain. Measurements were done in a 10 mM Tris-HCl buffer containing 1 mM EDTA and 1 M KCl with pH 8.0 at varying potentials. All nanopore data were recorded using a 250 kHz sampling rate with a 5 kHz low-pass filtering.

Supplementary Note 4

The event frequency, defined by dividing the cumulative blockage counts by the recording time[3], depends on the concentration of the analyte molecules in the *cis* compartment[4]. Voltage-dependent linear increase of the event frequency and the exponential decay of dwell time imply the electrokinetic translocation behaviors of 6SL-MPB passing through AeL ((Supplementary Figs. 12 b and c), according to the previous report[5]. Meanwhile, we also found the increase of the I_b/I_0 of the PIII domain and decrease of the percentage of the PIII domain in the whole events with the increase of applied voltage (Supplementary Fig. 12d), which is consistent with the translocation behavior of the short single-strand DNA (5′-ACTG-3′)[6]. This result suggests that the PIII domain was caused by the translocation of 6SL-MPB, where the increasing voltage accelerated the translocation through AeL nanopore but reduced the translocation probability. In addition, the voltage-dependent increase of the I_b/I_0 value of the PIII domain can be ascribed to the strengthened electroosmotic flow with increase of applied voltage, which decreased the Debye length (*i.e.*, thickness of electrical double layer) and thus increased the open current by increasing the effective pore size[7].

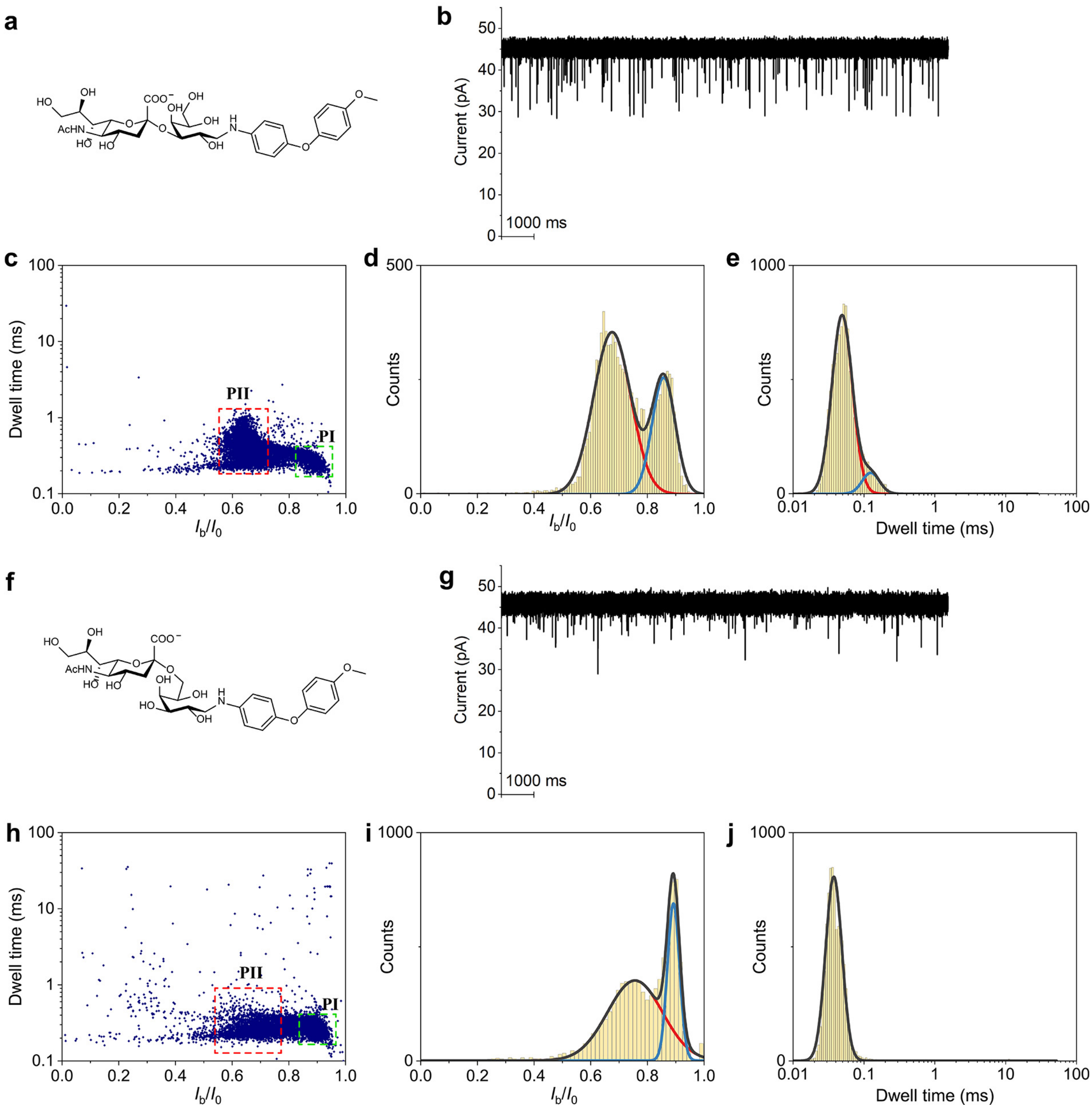

Supplementary Fig. 13. AeL nanopore test results of 3SG-MPB and 6SG-MPB. **a-e**, Chemical structure of 3SG-MPB (**a**), and the representative current traces (**b**), scatter plot of I_b/I_0 *vs.* dwell time (**c**), and the corresponding distribution of I_b/I_0 (**d**) and dwell time (**e**) from AeL nanopore measurement of 3SG-MPB. **f-j**, Chemical structure of 6SG-MPB (**f**), and the representative current traces (**g**), scatter plot of I_b/I_0 *vs.* dwell time (**h**), and distribution of I_b/I_0 (**i**) and dwell time (**j**) from AeL nanopore measurement of 6SG-MPB. Measurements were done in a 10 mM Tris-HCl buffer containing 1 mM EDTA and 1 M KCl with pH 8.0 at +100 mV. Nanopore data were recorded using a 250 kHz sampling rate with a 5 kHz low-pass filtering. Each scatter plot contains at least 9,000 events.

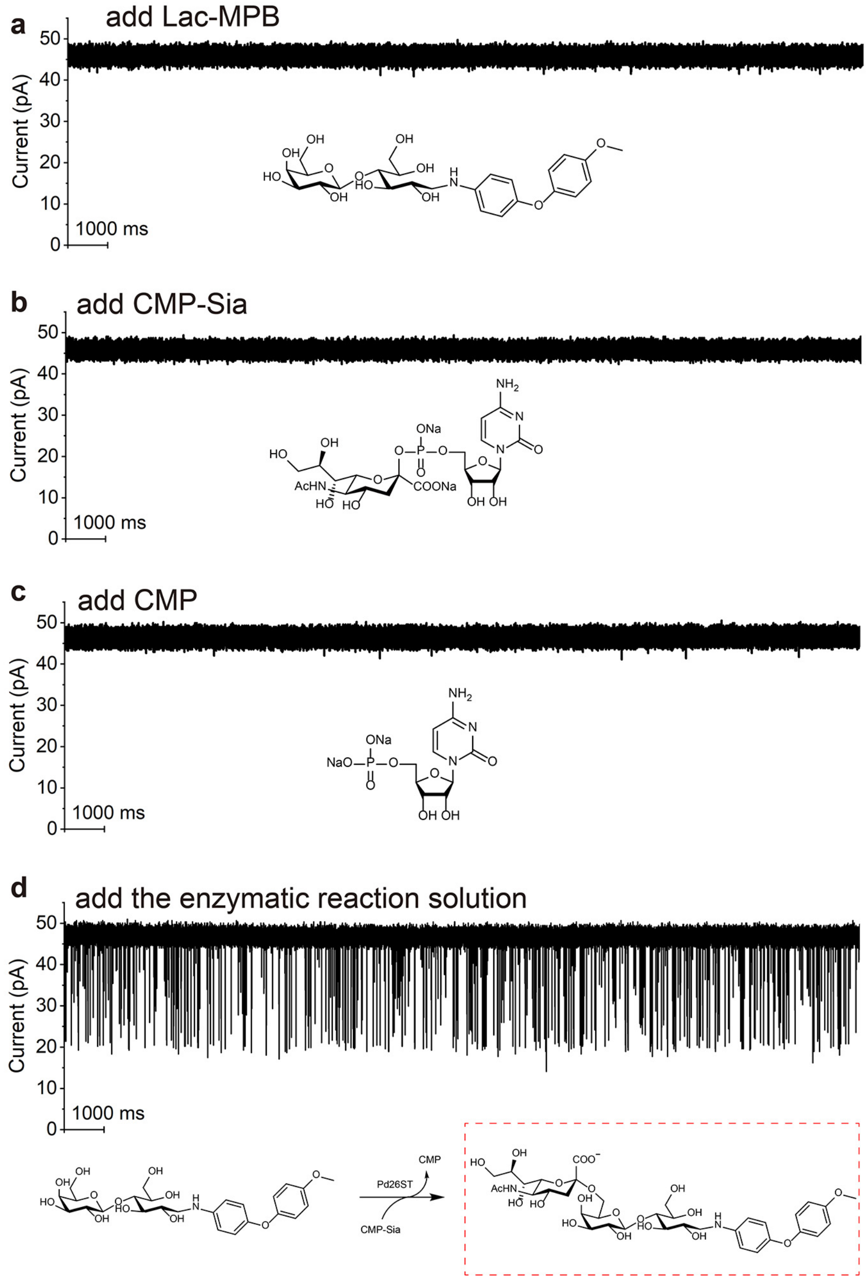

Supplementary Fig. 14. WT AeL nanopore detection of α2–6 sialylation of Lac-MPB with Pd26ST. **a-c**, The representative current trances from control experiments upon addition of Lac-MPB (**a**), CMP-Sia (**b**), and CMP (**c**). **d**, The representative current trances from the enzymatic reaction solution. Measurements were done in a 10 mM Tris-HCl buffer containing 1 mM EDTA and 1 M KCl with pH 8.0 at +100 mV. Nanopore data were recorded using a 250 kHz sampling rate with a 5 kHz low-pass filtering.

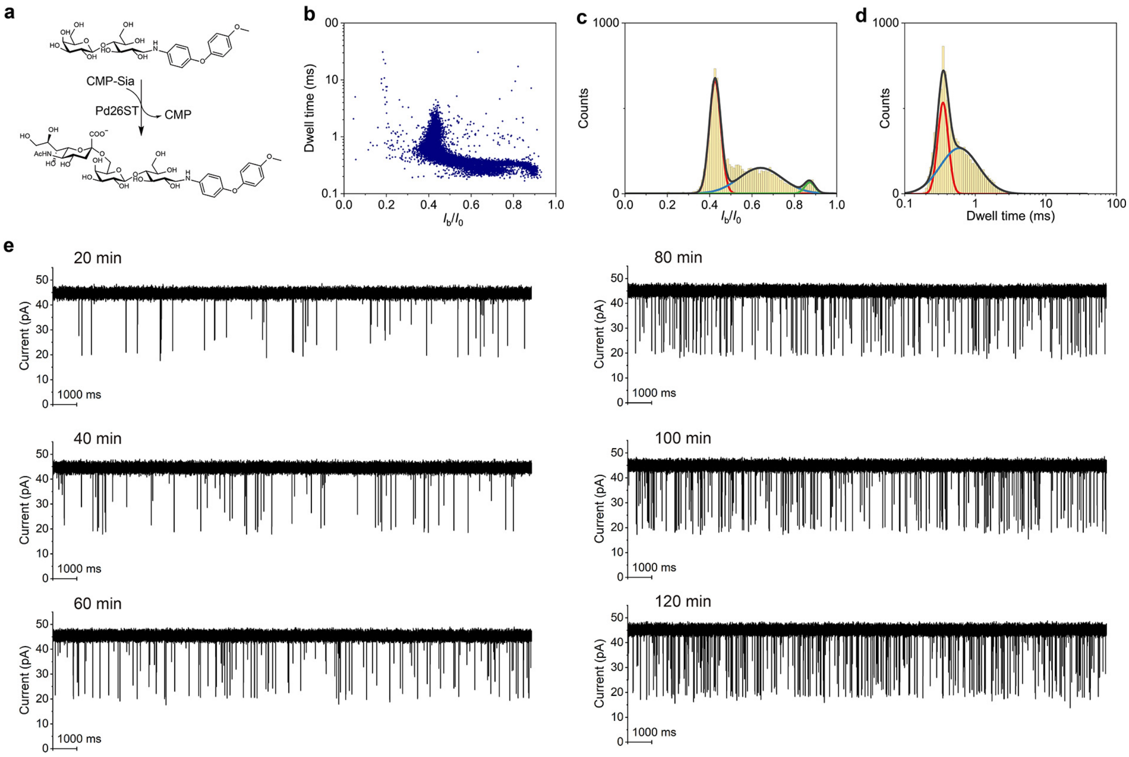

Supplementary Fig. 15. AeL nanopore detection of α2–6 sialylation of Lac-MPB. **a**, Schematic of α2–6 sialylation of Lac-MPB with Pd26ST. **b-d**, The event scatter plot (**b**), and its distribution in I_b/I_0 (**c**) and dwell time (**d**) dimension. The event characteristics of from the α2–6 sialylation reaction products are consistent with those from the individual 6SL-MPB. **e**, The representative current traces of AeL nanopore upon addition of the Pd26ST-catalyzed sialylation reaction solution from Lac-MPB substrate after a given period of reaction time, in which Lac-MPB was used as the substrate. Measurements were done in a 10 mM Tris-HCl buffer containing 1 mM EDTA and 1 M KCl with pH 8.0 at +100 mV. Nanopore data were recorded using a 250 kHz sampling rate with a 5 kHz low-pass filtering. Scatter plot contains at least 9,000 events. In order to avoid the effect for the reaction rate from low temperature (~24 °C) and high ion strength (1 M KCl) in nanopore test compartment (*i.e.*, the *cis* compartment), the sialylation reaction proceeds in a centrifuge tube in a thermal shaker with temperature of ~37 °C. Every 20 minutes 1 µL reaction solution was withdrawn and inactivated, and then injected into the *cis* solution of the new-assembled AeL nanopore measurement set-up. Correspondingly, the recorded blockage signal frequency correlates positively with the concentration of the sialylated product (*i.e.*, 6SL-MPB) at a specific point in time.

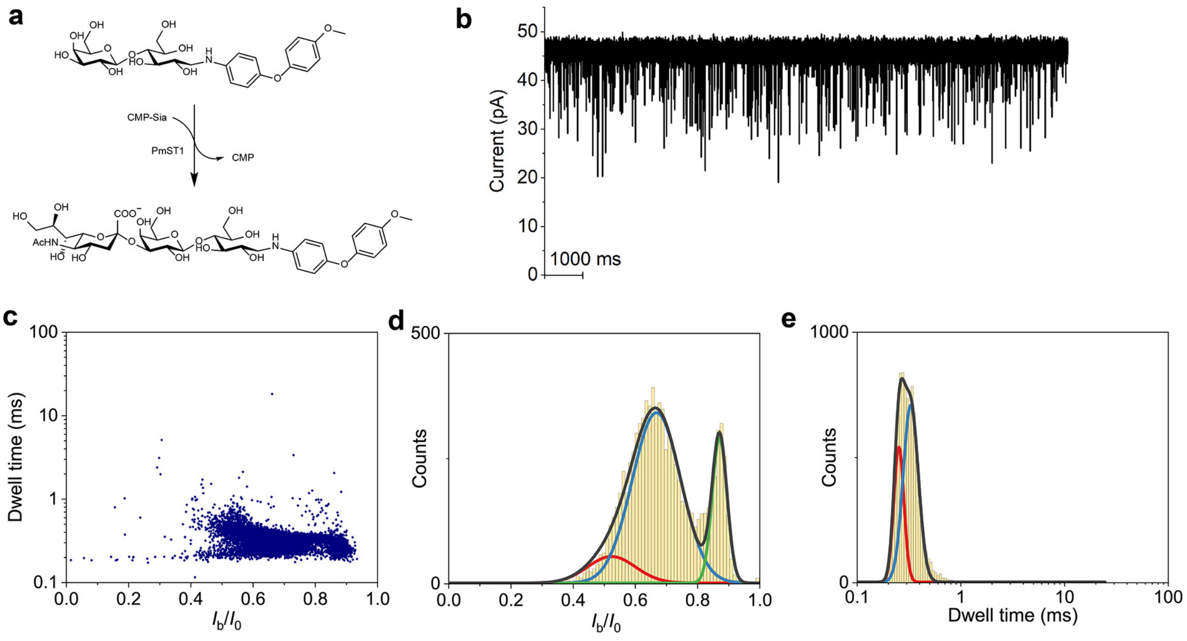

Supplementary Fig. 16. AeL nanopore detection of α2–3 sialylation of Lac-MPB with PmST1. **a**, Schematic of α2–3 sialylation of Lac-MPB catalyzed by PmST1 sialyltransferase. **b**, The representative current traces from the enzymatic reaction solution. **c-e**, The event scatter plot (**c**), and its distribution in I_b/I_0 (**d**) and dwell time (**e**) dimension. Measurements were done in a 10 mM Tris-HCl buffer containing 1 mM EDTA and 1 M KCl with pH 8.0 at +100 mV. Nanopore data were recorded using a 250 kHz sampling rate with a 5 kHz low-pass filtering. Scatter plot contains at least 9,000 events.

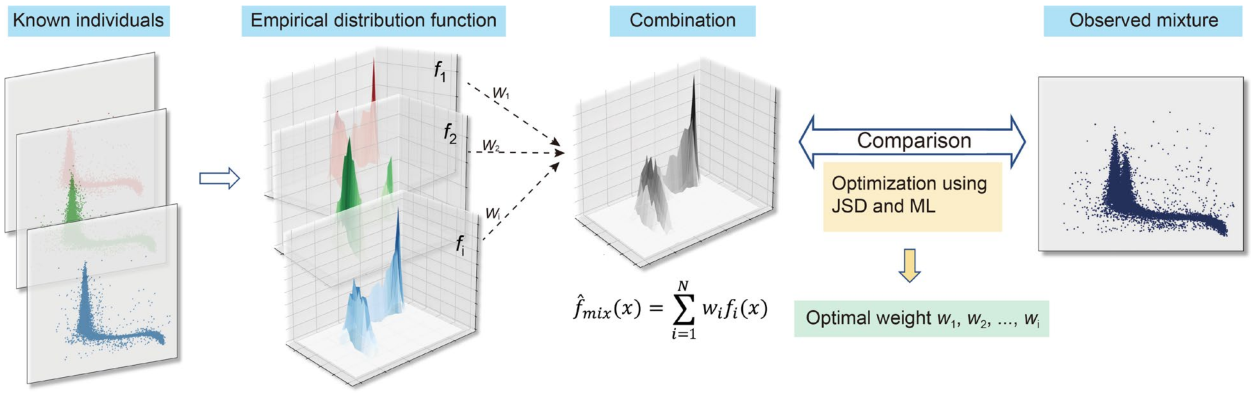

Supplementary Fig. 17. Schematic illustrating the flow chart of glycan identification and weight calculation results. The procedure is based on the comparison of the empirical distribution functions between virtual nanopore event combination and the observed mixture by optimizing the JSD using the machine learning methodology. Glycan weight calculation results from the developed automatic algorithm procedure.

a b c

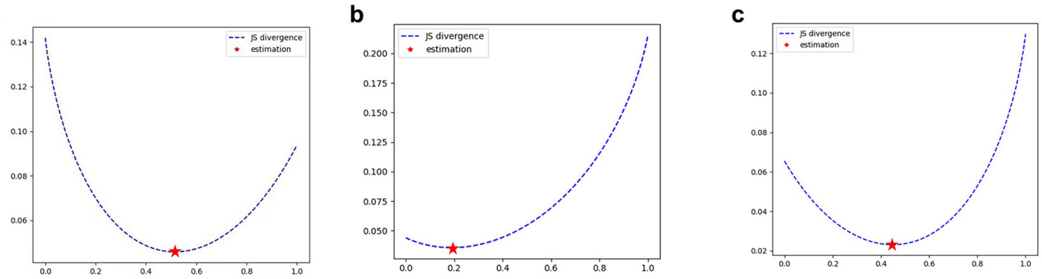

d e

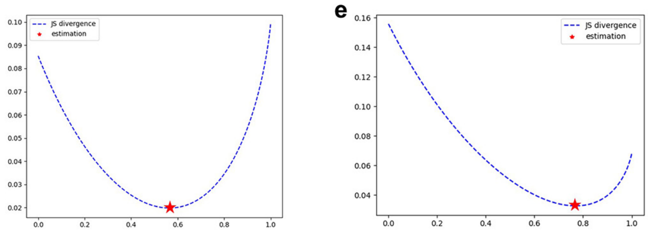

f

	Ratio or weight of 3SL-MPB in mixture				
Actual	0.2	0.4	0.5	0.6	0.8
Predicted	0.1894	0.4493	0.5155	0.5645	0.7696
JSD	0.035598	0.023043	0.045893	0.019830	0.032554

Supplementary Fig. 18. The results from MIX-distribution. **a-e**, The optimized JSD curves by comparing the empirical distribution functions between the possible combination and the observed binary mixtures of 3SL-MPB and 6SL-MPB with 3SL-MPB ratios of 0.5 (**a**), 0.2 (**b**), 0.4 (**c**), 0.6 (**d**), 0.8 (**e**). **f**, The predicted optimal weight of 3SL-MPB in various binary mixtures according the minimum JSD value.

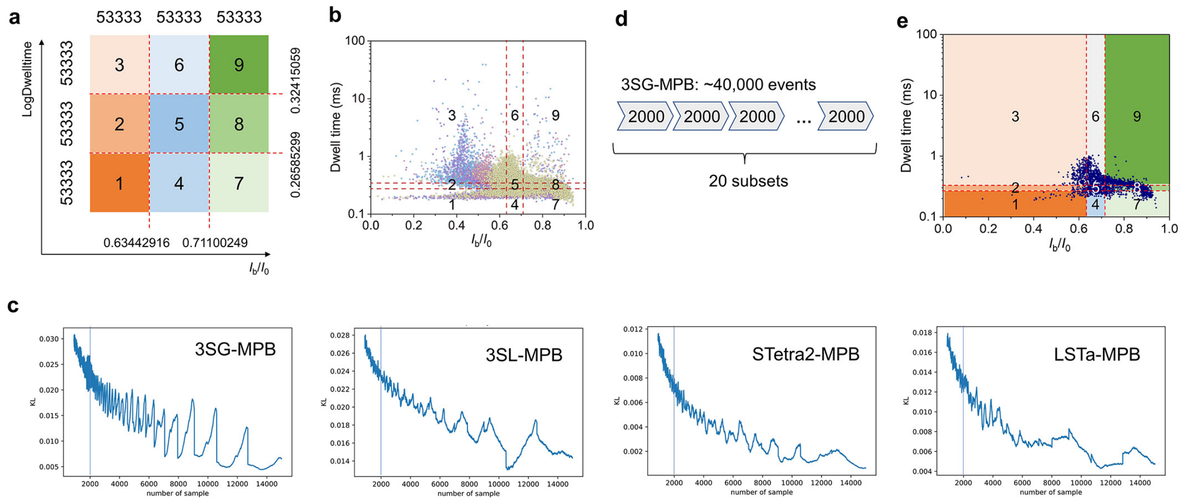

Supplementary Fig. 19. Nanopore data process and feature extraction for ML use. **a**,**b**, All events (~16,000) from four glycans (3SG-MPB, 3SL-MPB, STetra2-MPB, and LSTa-MPB) were binned into 9 domains using equal frequency binning in I_b/I_0 and dwell time dimension. **c**, Measurement of KL divergence to evaluate the similarity between the distribution of subset events with varied event number (ranging from 1000 events to 15000 events in a subset) and overall events. The mean KL value from the comparison of all subsets with equal event number with overall event of a glycan was plotted against subset size (*i.e.*, event number of subset). Blue lines indicate the final subset with 2000 events was determined. **d**, Taking 3SG-MPB as an example, all events were divided into 20 subsets, each of which contains 2000 events. **e**, An events subset of 3SG-MPB was partitioned into 9 domains with the above boundaries.

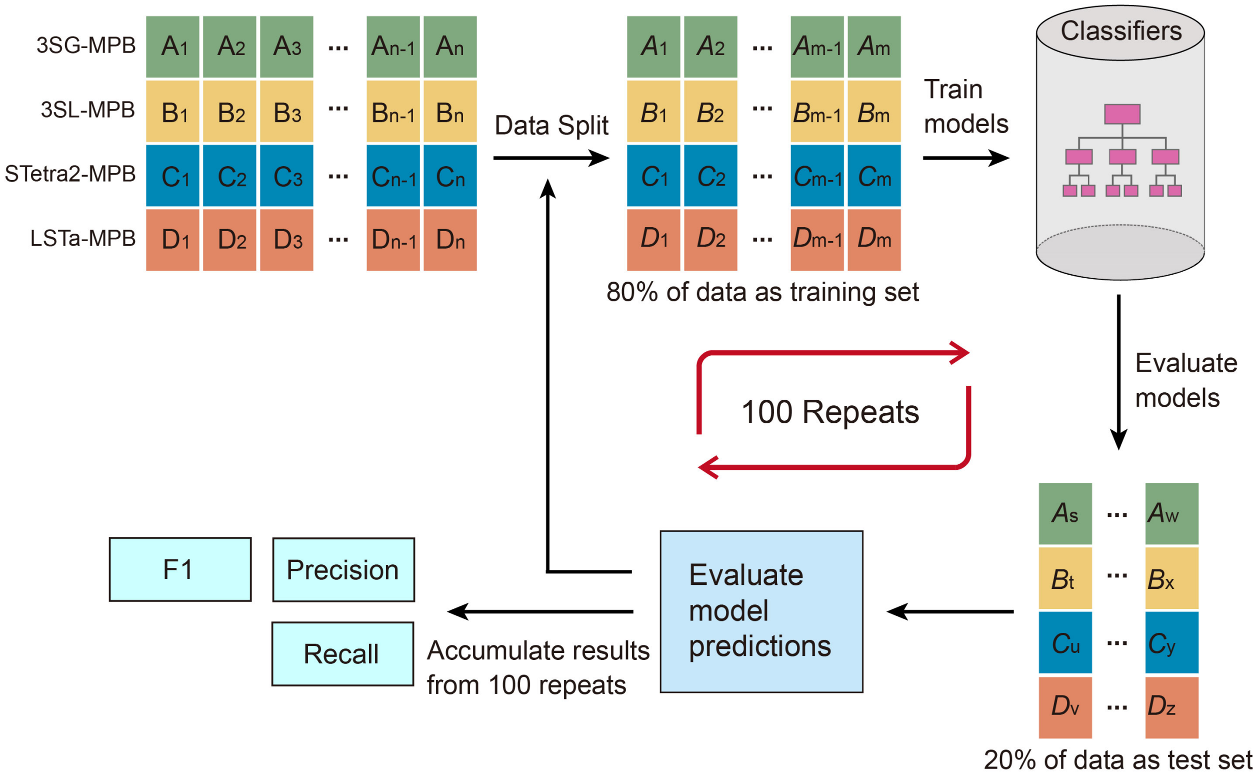

Supplementary Fig. 20. Schematic illustrating the flow chart of ML-based classification approach, where all event subsets of four glycans was split through a stratified random sampling method.

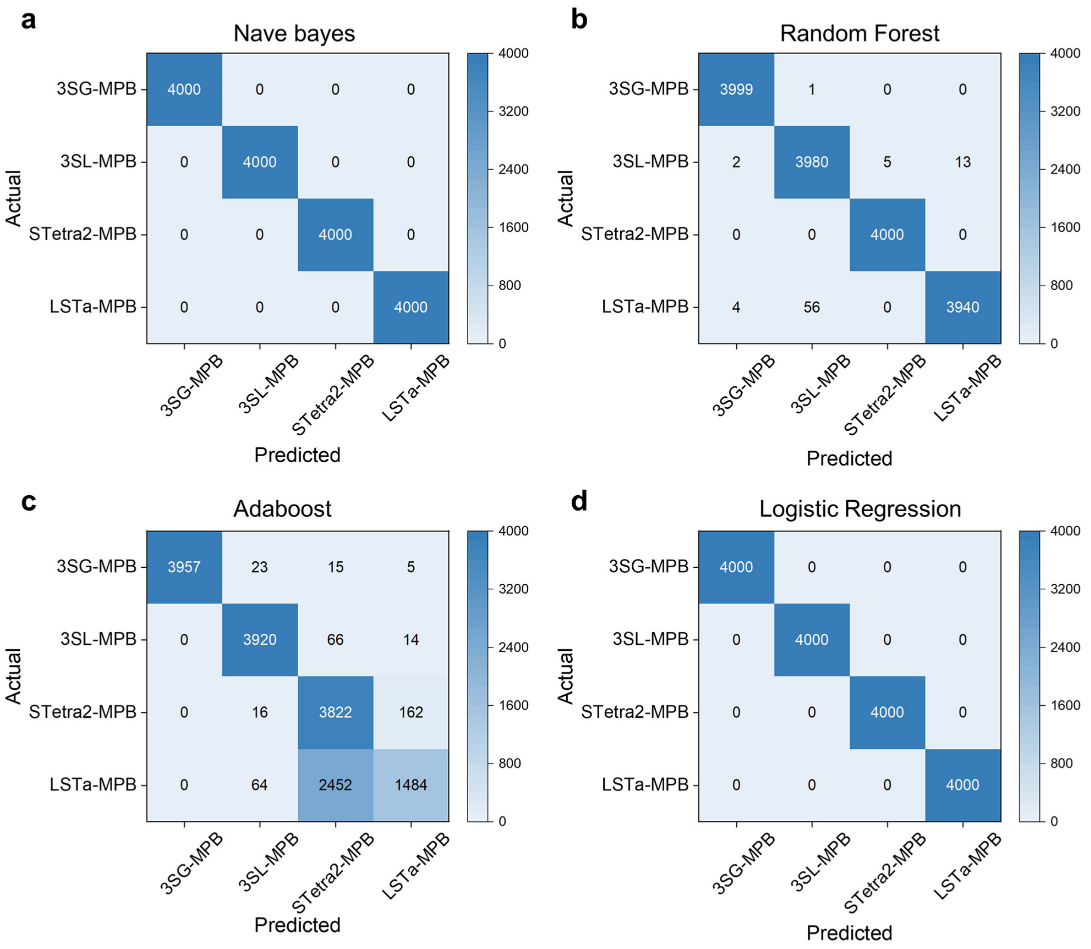

Supplementary Fig. 21. Confusion matrixes of other four models from the cumulative results of all testing sets towards identification of four glycans (3SG-MPB, 3SL-MPB, STetra2-MPB, and LSTa-MPB).

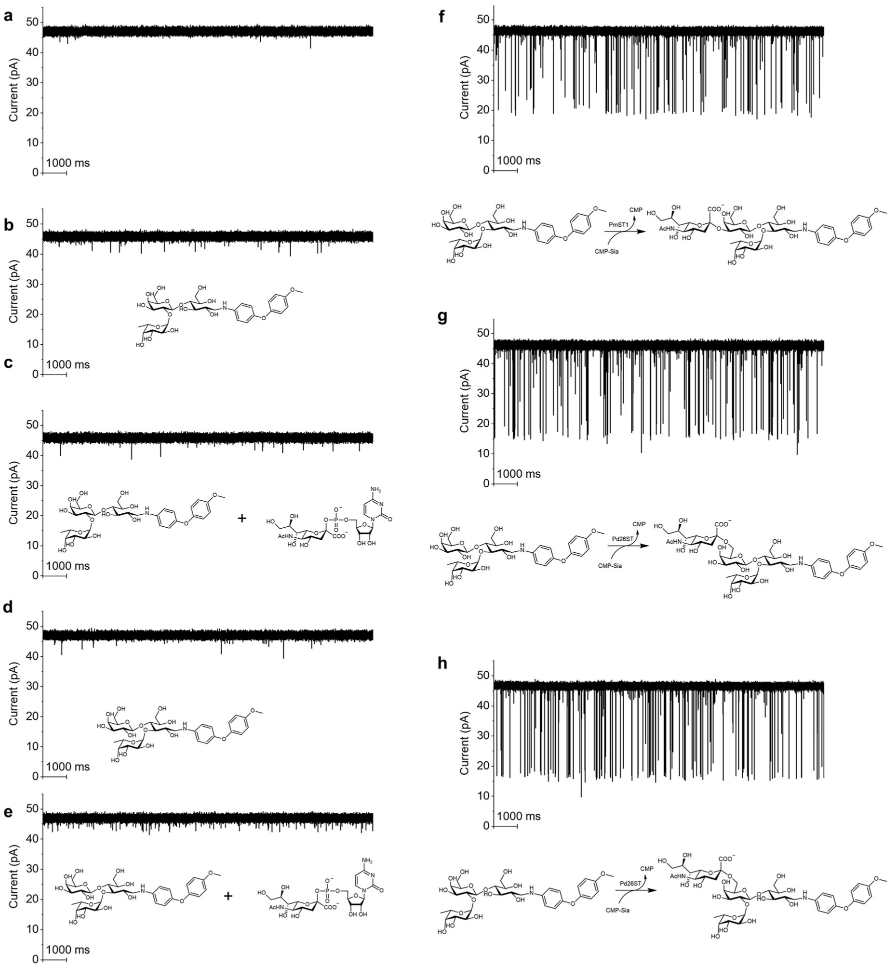

Supplementary Fig. 22. Enzymatic synthesis of three sialylglycans tagged with MPB (3S3FL-MPB, 6S3FL-MPB, and 6S2FL-MPB). **a**, Open current of AeL nanopore at +100 mV. **b-h**, The representative current traces of AeL nanopore from 2FL-MPB (**b**), the substrate solution (with 2FL-MPB and CMP-Sia) without sialyltransferase (**c**), 3FL-MPB (**d**), and the substrate solution (with 3FL-MPB and CMP-Sia) without sialyltransferase (**e**), the enzymatic reaction solutions on the substrate 3FL-MPB with PmST1 (**f**), 3FL-MPB with Pd26ST (**g**), and 2FL-MPB with Pd26ST (**h**). Insets of (**b-e**) are the corresponding chemical structure of main components that were added into the *cis* solution of AeL nanopore measurement set-up. Insets of (**f-h**) are the corresponding enzymatic reaction equations. Measurements were done in a 10 mM Tris-HCl buffer containing 1 mM EDTA and 1 M KCl with pH 8.0 at +100 mV. Nanopore data were recorded using a 250 kHz sampling rate with a 5 kHz low-pass filtering. According to above results, the substrate 2FL-MPB and 3FL-MPB cannot produce the obvious and identifiable blockage signals, the observed blockage signals when adding the enzymatic reaction solution can be ascribed to the blockages caused by the corresponding product–sialylglycans. Thus, in the following section, the enzymatic reaction solution was directly used for the nanopore test to provide the blockage signals the corresponding sialylglycan.

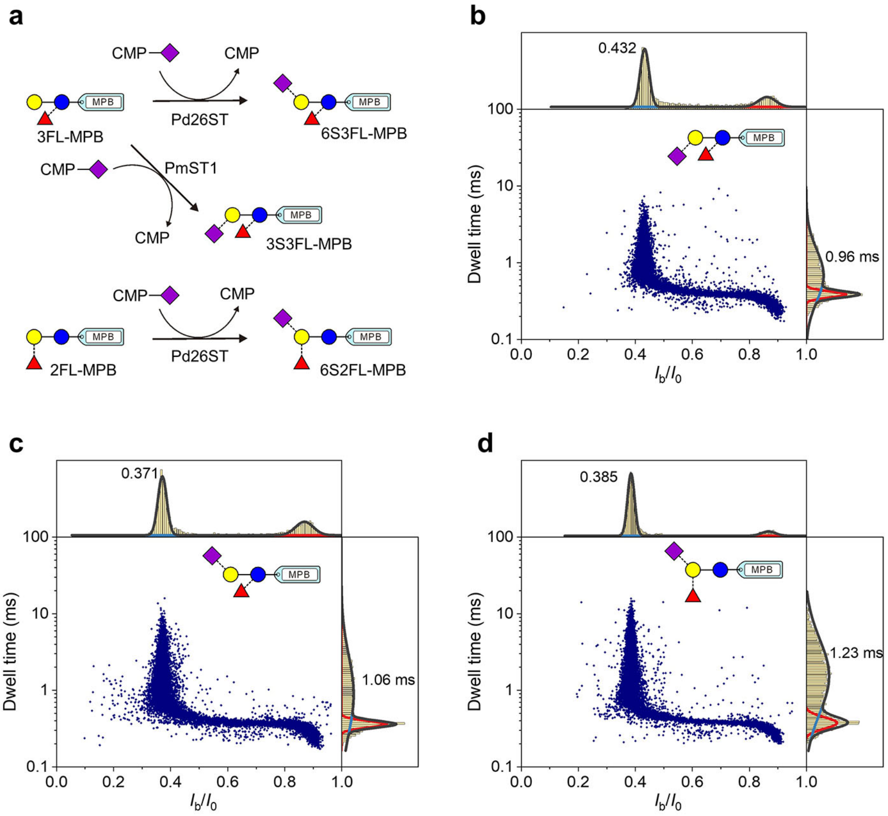

Supplementary Fig. 23. Enzymatic synthesis of three sialylglycans tagged with MPB (3S3FL-MPB, 6S3FL-MPB, and 6S2FL-MPB). **a** Schematic illustrating the enzymatic synthesis. **b-d** The scatter plots and distributions in I_b/I_0 and dwell time from blockage events of AeL nanopore measurements of three glycans. I_b/I_0 and dwell time distributions of the characteristic population were marked with the mean values from Gaussian fitting. Insets of (**b-d**) are the chemical structures of enzymatic products. All measurements were done in a 10 mM Tris-HCl buffer containing 1 mM EDTA and 1 M KCl with pH 8.0 at +100 mV. All nanopore data were recorded using a 250 kHz sampling rate with a 5 kHz low-pass filtering. Each scatter plot contains at least 9,000 events.

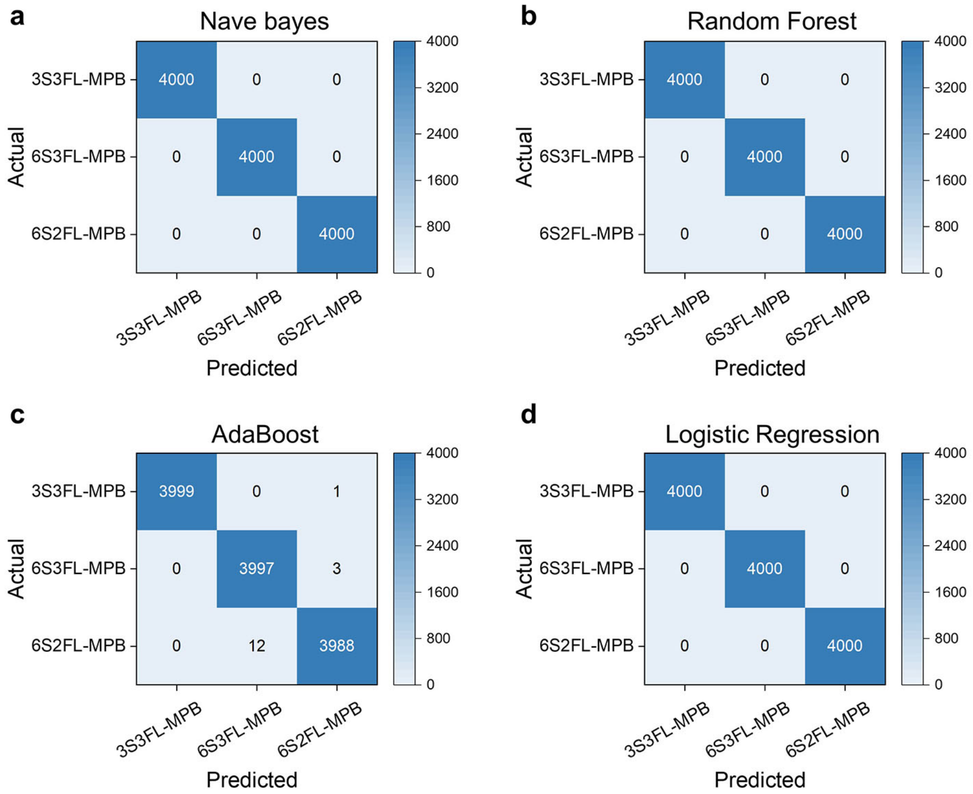

Supplementary Fig. 24. Confusion matrixes of other four models from the cumulative results of all testing sets towards identification of three sialylglycans with branched fucose (*i.e.*, 3S3FL-MPB, 6S3FL-MPB, and 6S2FL-MPB).

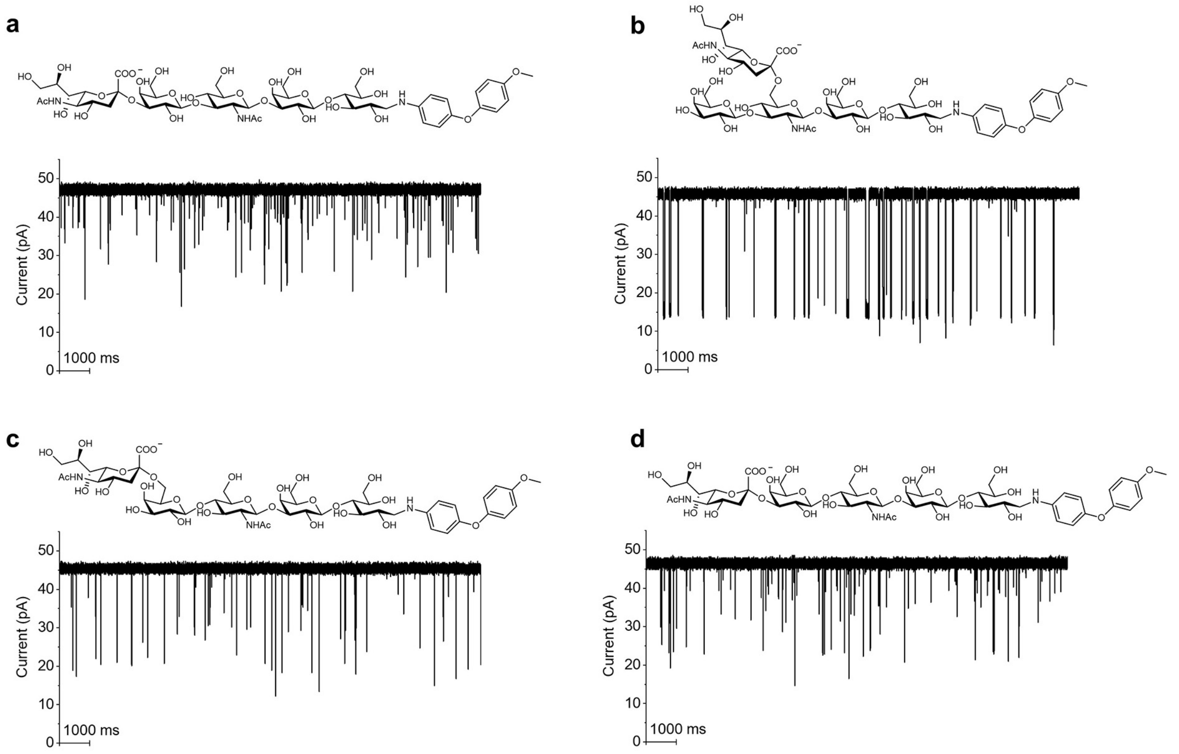

Supplementary Fig. 25. The chemical structure of LSTa-MPB (**a**), LSTb-MPB (**b**), LSTc-MPB (**c**), and LSTd-MPB (**d**) and the corresponding current trace from AeL nanopore measurements. All measurements were done in a 10 mM Tris-HCl buffer containing 1 mM EDTA and 1 M KCl with pH 8.0 at +100 mV. Nanopore data of LSTb-MPB was recorded using a 100 kHz sampling rate with a 5 kHz low-pass filtering. Other data were recorded using a 250 kHz sampling rate with a 5 kHz low-pass filtering.

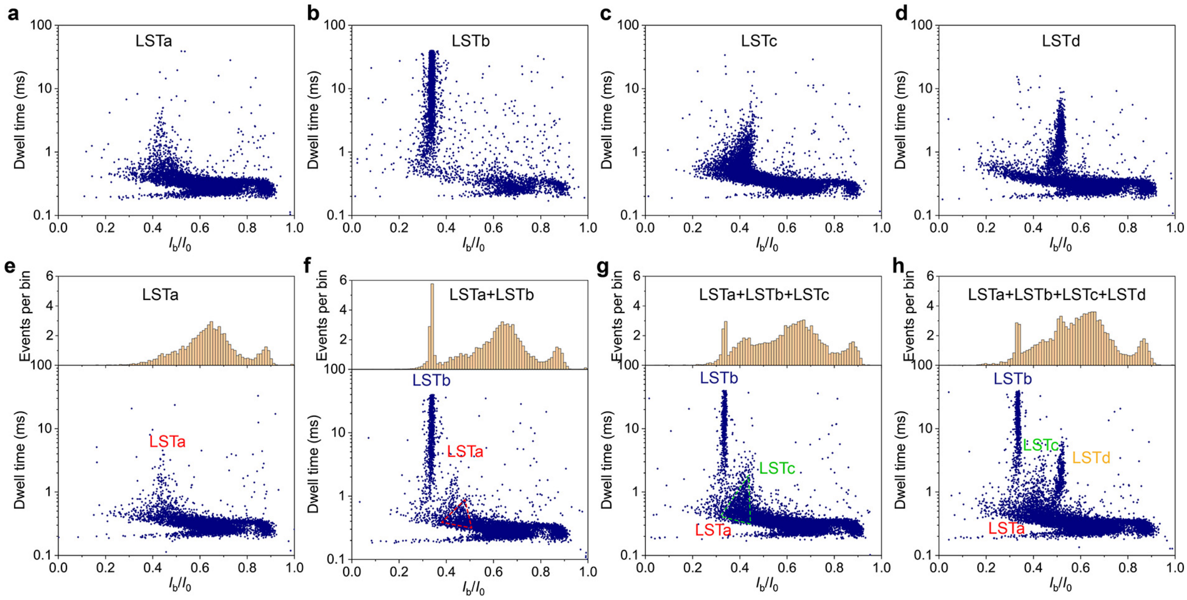

Supplementary Fig. 26. The nanopore test results of four pentasaccharide isomers (e.g., LSTa, LSTd, LSTc, and LSTb) with MPB tag. **a-d** Scatter plots of four pentasaccharide isomers. **e-h** Scatter plots and the corresponding I_b/I_0 distributions acquired when LSTa-MPB, LSTb-MPB, LSTc-MPB, and LSTd-MPB were sequentially added to the cis solution. All measurements were done in a 10 mM Tris-HCl buffer containing 1 mM EDTA and 1 M KCl with pH 8.0 at +100 mV. All nanopore data were recorded using a 250 kHz sampling rate with a 5 kHz low-pass filtering.

Supplementary Note 5

The nanopore test results (Supplementary Fig. 26) of four pentasaccharide isomers (e.g., LSTa, LSTd, LSTc, and LSTb) with MPB tag show the distinct difference among them. Furthermore, we designed a measurement towards the mixtures of these glycans based on a same AeL nanopore by adding the glycan sample into *cis* solution in sequence. The results show that LSTb and LSTd were readily recognized in different mixtures due to their prominent characteristic. As for LSTa and LSTc, these two glycans can be roughly recognized when the mixture contain fewer samples, for example, LSTa-LSTb mixture and LSTa-LSTb-LSTc mixture. When the mixture contains all four glycans, LSTa and LSTc are basically indistinguishable due to severe superposition of scatter plots.

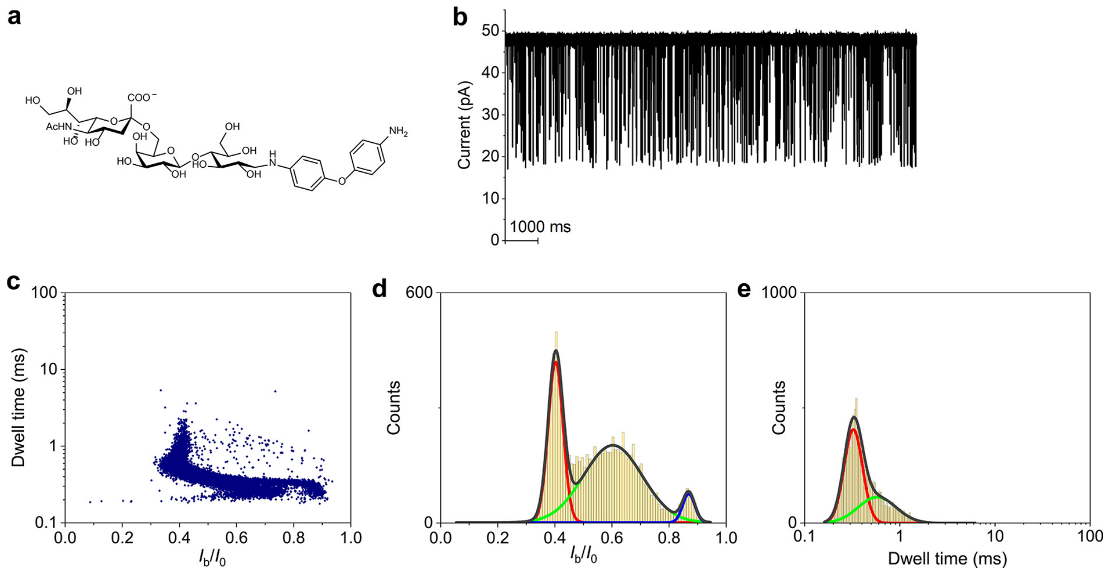

Supplementary Fig. 27. Nanopore test results of 6SL-DPE-NH_2. **a-e**, The chemical structure of 6SL-DPE-NH_2 (**a**), and the nanopore current traces (**b**), scatter plot of I_b/I_0 *vs.* dwell time (**c**), and the corresponding distribution of I_b/I_0 (**d**) and dwell time (**e**) from AeL nanopore measurement of 6SL-DPE-NH_2. Measurement was done in a 10 mM Tris-HCl buffer containing 1 mM EDTA and 1 M KCl with pH 8.0 at +100 mV. Nanopore data were recorded using a 250 kHz sampling rate with a 5 kHz low-pass filtering. Scatter plot contains at least 9,000 events.

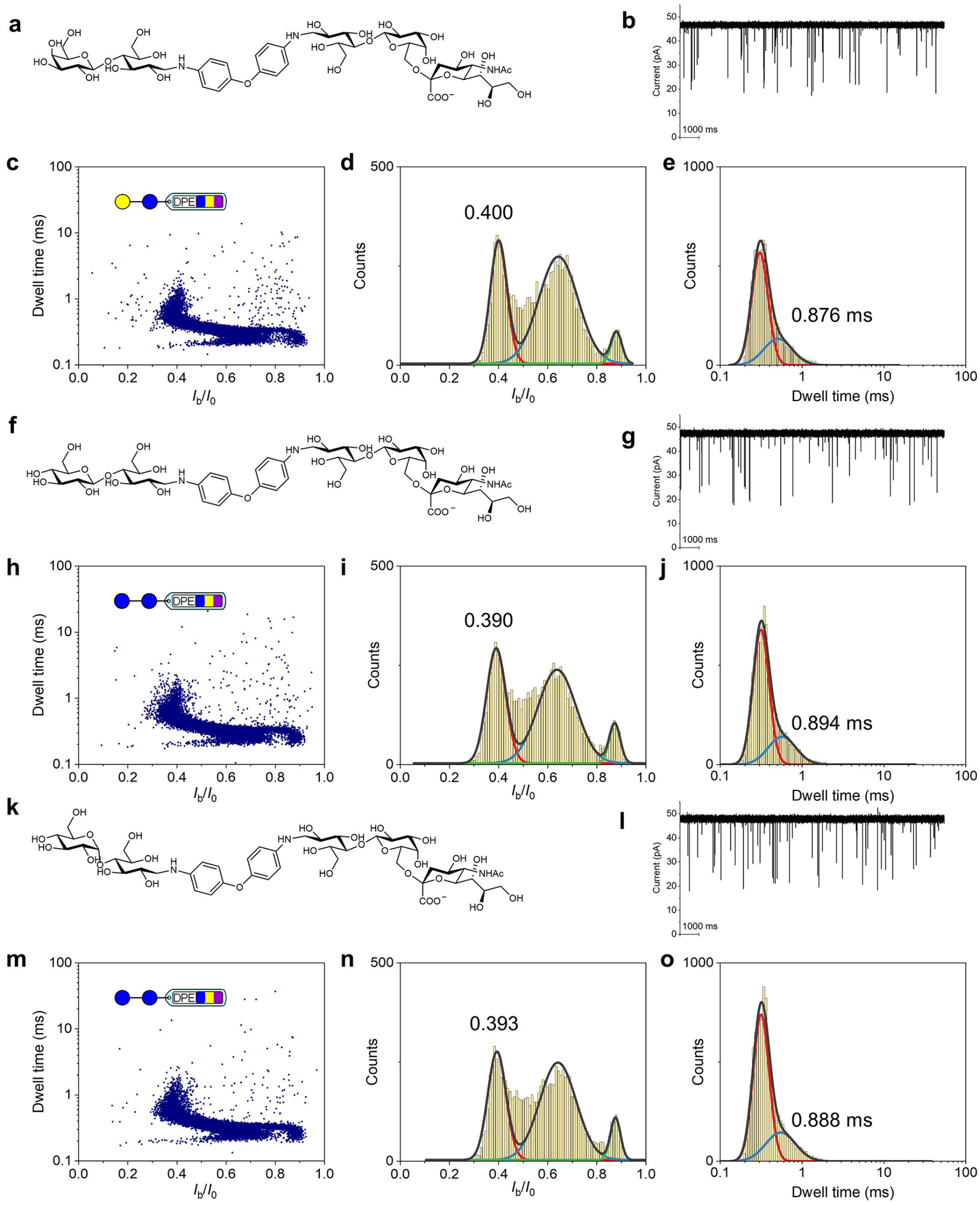

Supplementary Fig. 28. Nanopore test results of three disaccharide derivatives. **a-e**, The chemical structure of Lac-DPE-6SL (**a**), and the nanopore current traces (**b**), scatter plot of I_b/I_0 *vs.* dwell time (**c**), and the corresponding distribution of I_b/I_0 (**d**) and dwell time (**e**) from AeL nanopore measurement of Lac-DPE-6SL. **f-j**, The chemical structure of Cel-DPE-6SL (**f**), and the nanopore current traces (**g**), scatter plot of I_b/I_0 *vs.* dwell time (**h**), and the corresponding distribution of I_b/I_0 (**i**) and dwell time (**j**) from AeL nanopore measurement of Cel-DPE-6SL. **k-o**, The chemical structure of Mal-DPE-6SL (**k**), and the nanopore current traces (**l**), scatter plot of I_b/I_0 *vs.* dwell time (**m**), and the corresponding distribution of I_b/I_0 (**n**) and dwell time (**o**) from AeL nanopore measurement of Mal-DPE-6SL. All measurements were done in a 10 mM Tris-HCl buffer containing 1 mM EDTA and 1 M KCl with pH 8.0 at +100 mV. All nanopore data were recorded using a 250 kHz sampling rate with a 5 kHz low-pass filtering. Each scatter plot contains at least 9,000 events.

a

Model	Feature number	Subset size	F1	Precision	Recall
SVM	21^2	2500	0.8452	0.8763	0.8573
Native Bayes	17^2	2700	0.7312	0.7699	0.7632
Random Forest	12^2	2400	0.8307	0.8666	0.8437
AdaBoost	30^2	2500	0.935	0.9421	0.9122
Logistic Regression	11^2	2500	0.6905	0.6997	0.7398

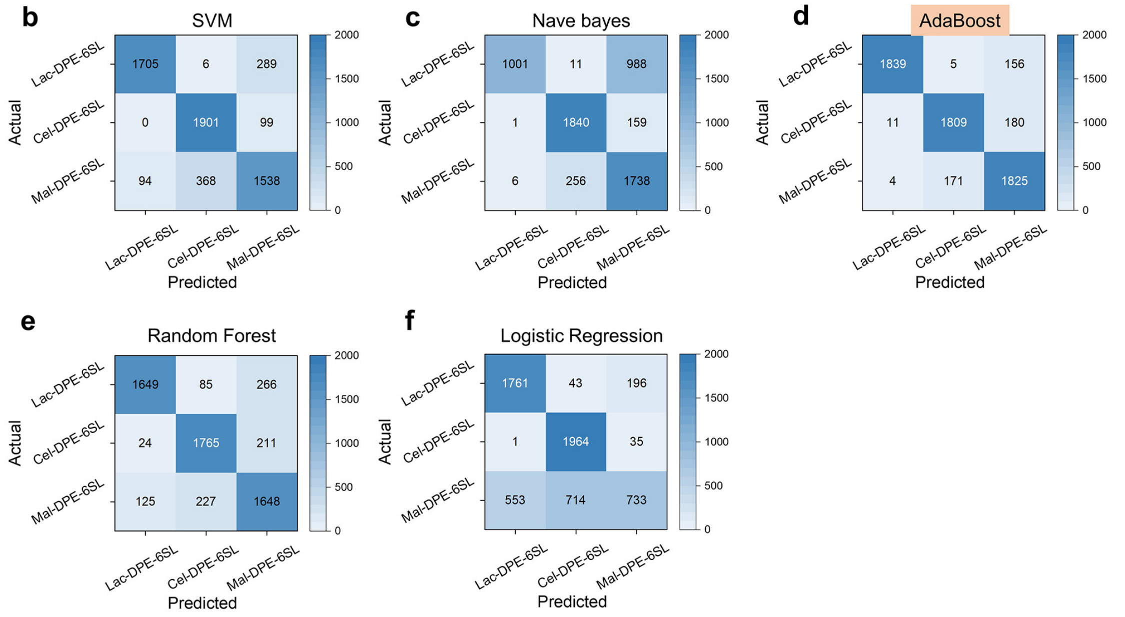

Supplementary Fig. 29. ML-based classification for three neutral disaccharide derivatives. **a**, Evaluation results of five models according to the optimized binning number and subset size for ML-based classification of three neutral disaccharide derivatives. **b-f**, Confusion matrixes of five models from the cumulative results of all testing sets after 10 replicates towards identification of three neutral disaccharide derivatives (Lac-DPE-6SL, Cel-DPE-6SL, Mal-DPE-6SL) based on the optimized binning number and subset size. Based on the previous binning number and subset size, all the models only gave the moderate accuracy. Further, by increasing the bin number and optimizing the subset size, the resulting classifiers show the improved accuracies. And the classifier based on AdaBoost model reported the highest accuracy of up to 96.34% (F1 score), which accurately predicted most of test sets. This suggests the clear discrimination of three disaccharides, further demonstrating the power of the integration of nanopore sensing with machine learning method towards the identification of glycan stereoisomers.

a

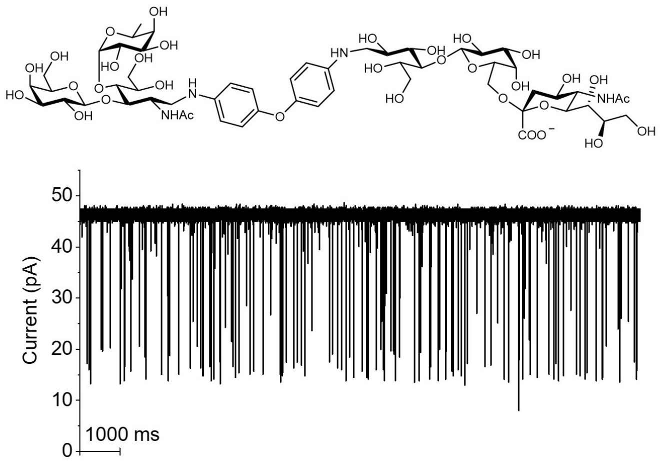

b

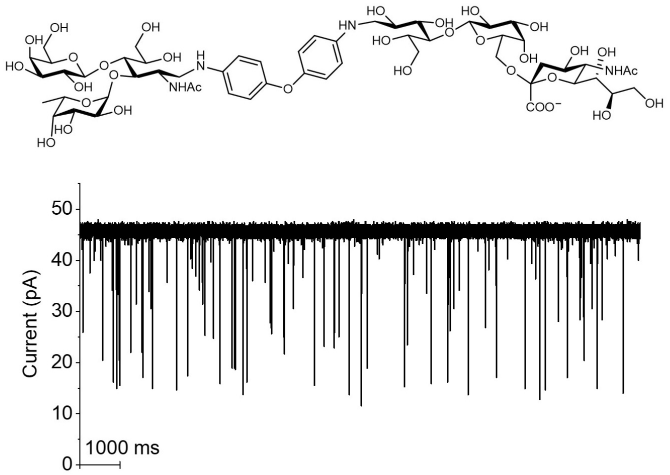

Supplementary Fig. 30. AeL nanopore test results of a pair of neutral branched trisaccharide derivatives. **a**, The chemical structure of LeA-DPE-6SL and its representative nanopore ionic current traces. **b**, The chemical structure of LeX-DPE-6SL and its representative nanopore ionic current traces. The final concentrations of these two glycan derivatives in the *cis* solution are approximately 10 μM. Both measurements were done in a 10 mM Tris-HCl buffer containing 1 mM EDTA and 1 M KCl with pH 8.0 at +100 mV. Nanopore data were recorded using a 250 kHz sampling rate with a 5 kHz low-pass filtering.

a

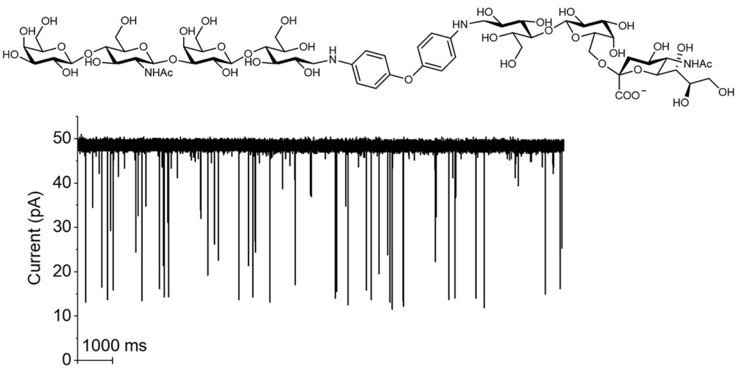

b

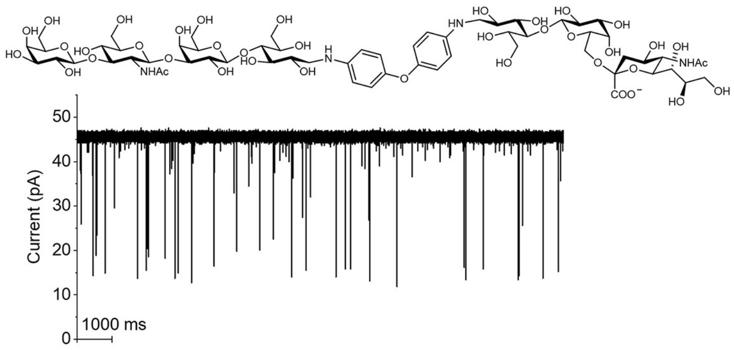

Supplementary Fig. 31. AeL nanopore test results of a pair of neutral tetrasaccharide derivatives. **a**, The chemical structure of LNnT-DPE-6SL and its representative nanopore ionic current traces. **b**, The chemical structure of LNT-DPE-6SL and its representative nanopore ionic current traces. The final concentrations of these two glycan derivatives in the *cis* solution are approximately 10 μM. Both measurements were done in a 10 mM Tris-HCl buffer containing 1 mM EDTA and 1 M KCl with pH 8.0 at +100 mV. Nanopore data were recorded using a 250 kHz sampling rate with a 5 kHz low-pass filtering.

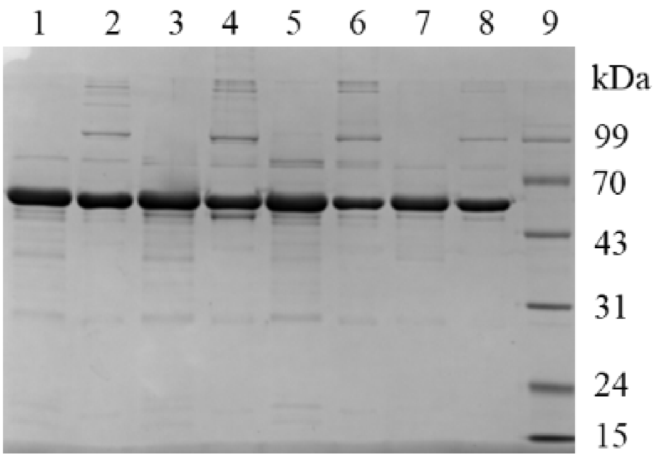

Supplementary Fig. 32. SDS PAGE analysis of proaerolysin mutants. 1: reduced R220A sample; 2: non-reduced R220A sample; 3: reduced K238A sample; 4: non-reduced K238A sample; 5: reduced K242A sample; 6: non-reduced K242A; 7: reduced R282A sample; 8: non-reduced R282A sample; 9: Marker. This experiment was repeated independently at least three times.

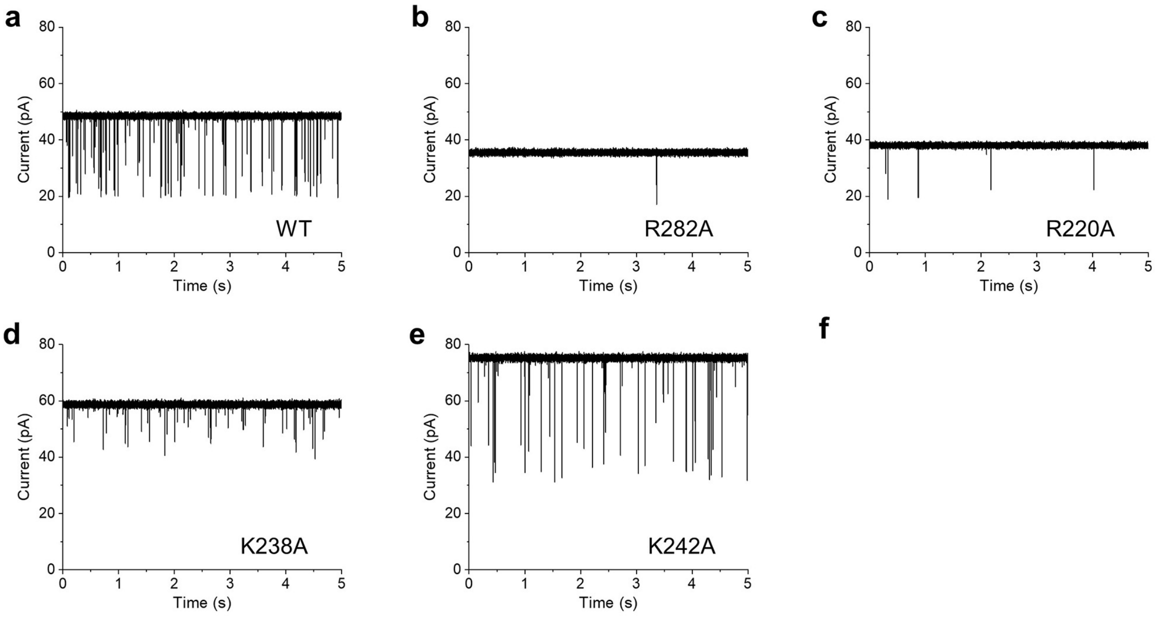

Supplementary Fig. 33. The representative nanopore ionic current traces of WT AeL and four mutants (**a-e**) in the presence of 2 μM 6SL-MPB at +100 mV. All measurements were done in a 10 mM Tris-HCl buffer containing 1 mM EDTA and 1 M KCl with pH 8.0 at +100 mV. Nanopore data were recorded using a 250 kHz sampling rate with a 5 kHz low-pass filtering.

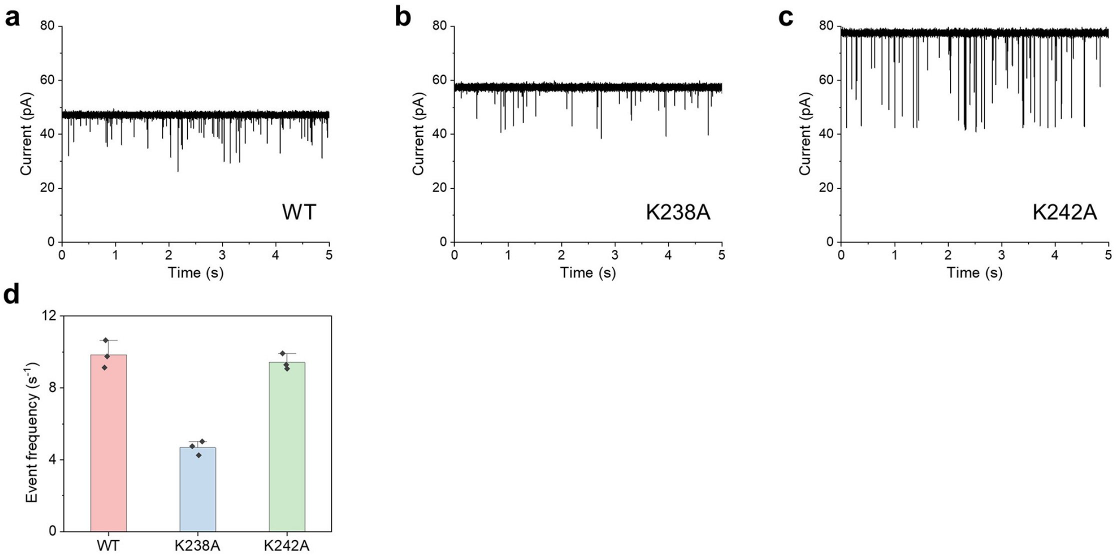

Supplementary Fig. 34. The representative nanopore ionic current traces of WT, K238A and K242A mutants (**a**-**c**) in the presence of 2 μM 3SL-MPB at +100 mV, and their event frequencies (**d**). Data are presented as bar charts (**d**) with mean values of frequency ± SD of three independent experiments. Individual data points are shown as black dots. All measurements were done in a 10 mM Tris-HCl buffer containing 1 mM EDTA and 1 M KCl with pH 8.0 at +100 mV. Nanopore data were recorded using a 250 kHz sampling rate with a 5 kHz low-pass filtering.

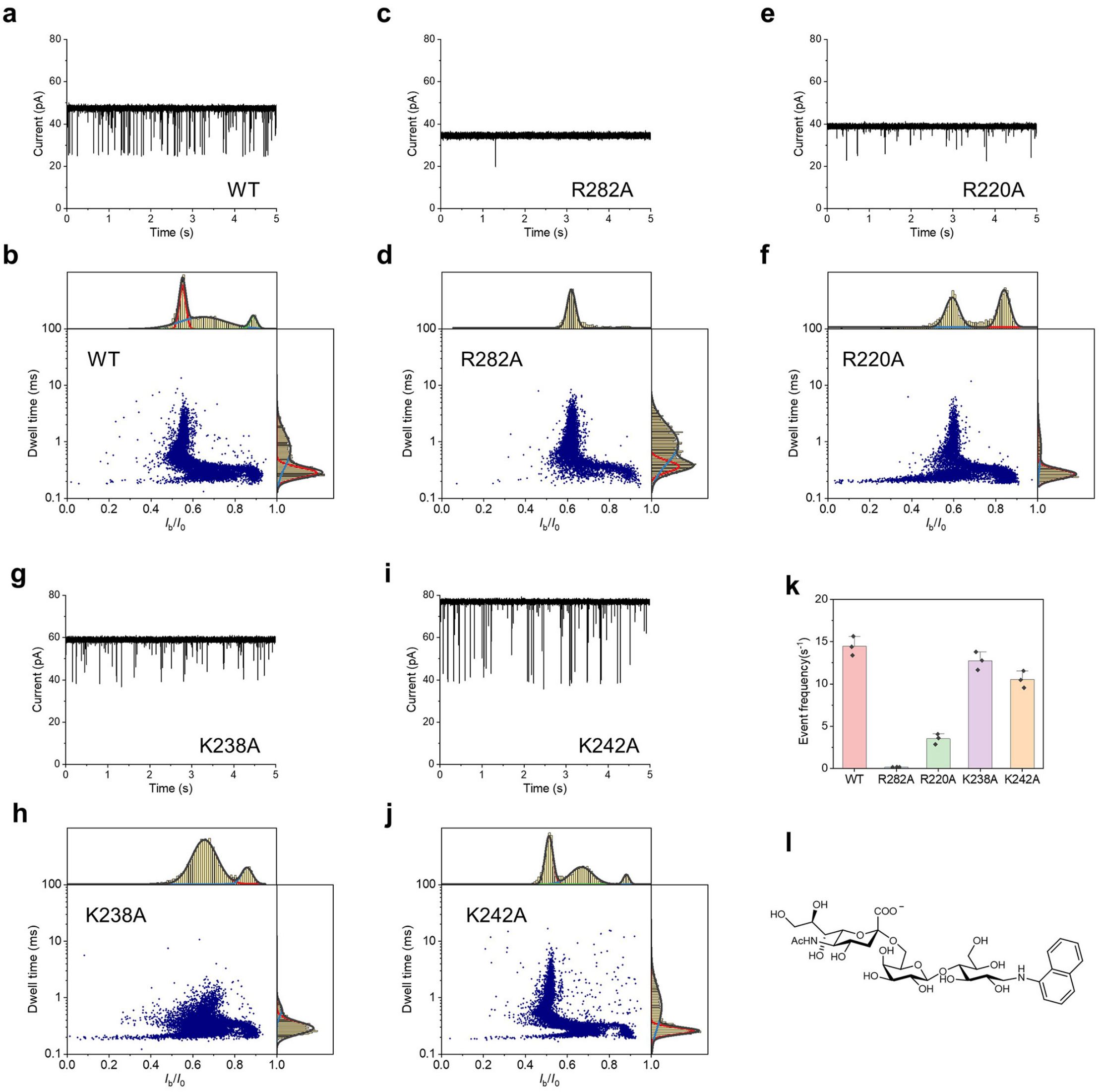

Supplementary Fig. 35. Test results of WT AeL and four mutants toward 6SL-NA. **a-j**, The representative current traces of WT AeL and four mutants in the presence of 2 μM 6SL-NA (**a**, **c**, **d**, **g**, and **i**) at +100 mV, and the corresponding event scatter plot and I_b/I_0 and dwell time distribution (**b**, **d**, **f**, **g**, and **j**). **k**, Comparison of event frequency of WT and four mutants in the presence of 2 μM 6SL-NA with same concentration. Data are presented as bar charts with mean values of frequency ± SD of three independent experiments. Individual data points are shown as black dots. **l**, Chemical structure of 6SL-NA. All measurements were done in a 10 mM Tris-HCl buffer containing 1 mM EDTA and 1 M KCl with pH 8.0 at +100 mV. Nanopore data were recorded using a 250 kHz sampling rate with a 5 kHz low-pass filtering. The scatter plot of R282A contains about 5000 events due to the ultra-low event frequency. Each of other scatter plot contains at least 9,000 events.

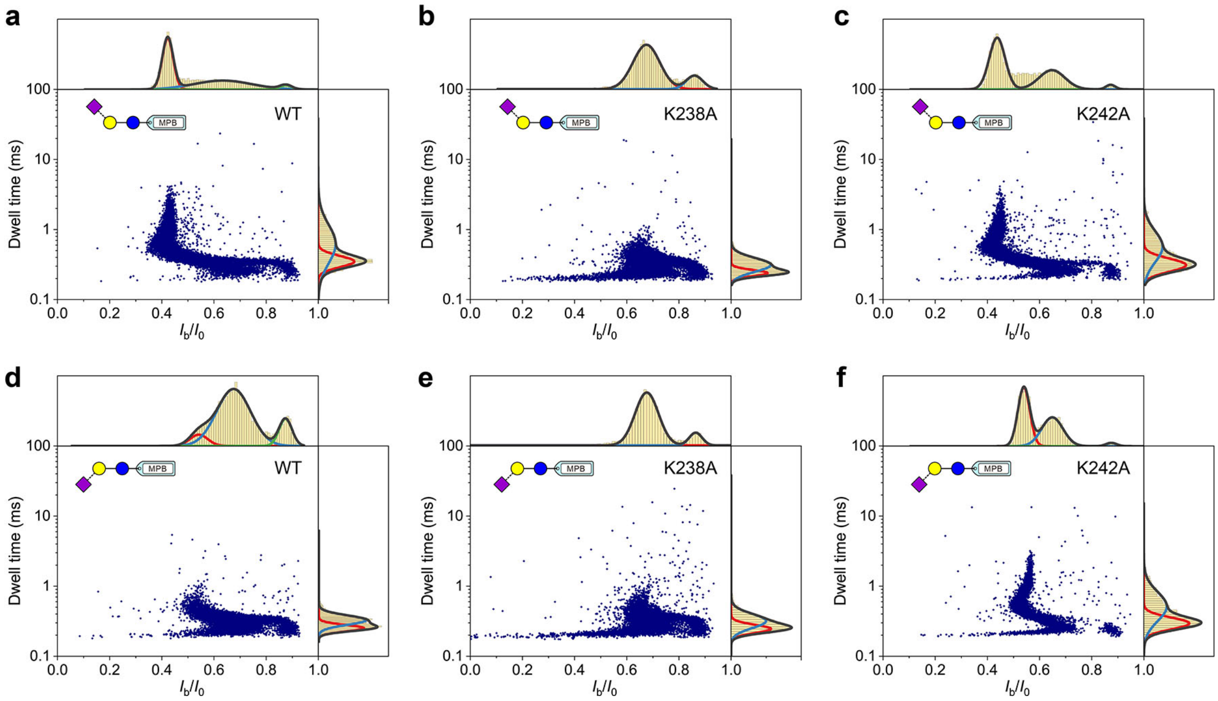

Supplementary Fig. 36. Nanopore data of WT AeL and two mutants towards 6SL-MPB (**a-c**) or 3SL-MPB (**d-e**). Event characteristics of 6SL-MPB and 3SL-MPB measured with WT AeL or K242A mutant exhibit remarkable difference. However, the event from K238A almost shows the identical population characteristic for these two glycans, indicating that the mutation K238A lost the crucial discerning ability for the glycan linkage isomers. All measurements were done in a 10 mM Tris-HCl buffer containing 1 mM EDTA and 1 M KCl with pH 8.0 at +100 mV. Nanopore data were recorded using a 250 kHz sampling rate with a 5 kHz low-pass filtering. Each scatter plot contains at least 9,000 events.

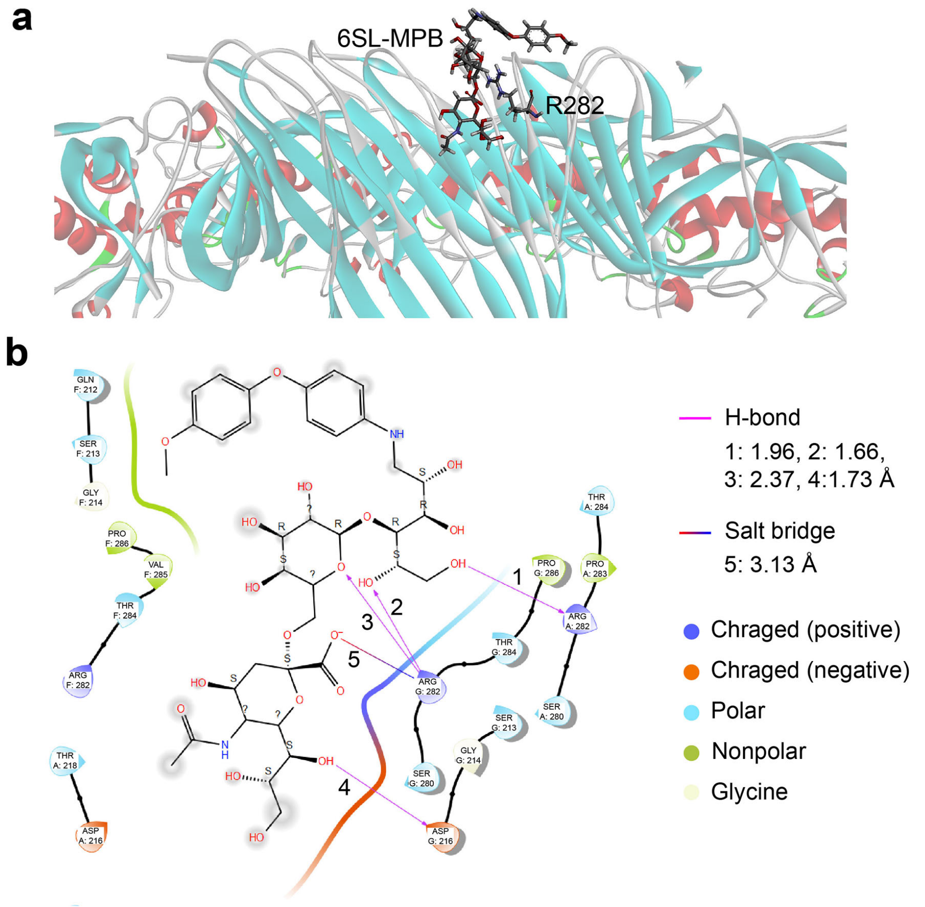

Supplementary Fig. 37. Interaction of 6SL-MPB with AeL in R282 zone. **a**, Partial interaction model from the molecular docking simulation using Schrödinger software suite. **b**, The detailed interaction pattern.

References

1. Pastoriza-Gallego, M., Thiébot, B., Bacri, L., Auvray, L. & Pelta, J. Dynamics of a polyelectrolyte through aerolysin channel as a function of applied voltage and concentration. *Eur. Phys. J. E* **41**, 58 (2018).

2. Chen, X., Zhang, Y., Mohammadi Roozbahani, G. & Guan, X. Salt-mediated nanopore detection of adam-17. *ACS Appl. Bio Mater.* **2**, 504-509 (2019).

3. Meng, F.-N., Ying, Y.-L., Yang, J. & Long, Y.-T. A wild-type nanopore sensor for protein kinase activity. *Anal. Chem.* **91**, 9910-9915 (2019).

4. Fennouri, A., Przybylski, C., Pastoriza-Gallego, M., Bacri, L., Auvray, L. & Daniel, R. Single molecule detection of glycosaminoglycan hyaluronic acid oligosaccharides and depolymerization enzyme activity using a protein nanopore. *ACS Nano* **6**, 9672-9678 (2012).

5. Cao, C., Ying, Y.-L., Hu, Z.-L., Liao, D.-F., Tian, H. & Long, Y.-T. Discrimination of oligonucleotides of different lengths with a wild-type aerolysin nanopore. *Nat. Nanotechnol.* **11**, 713-718 (2016).

6. Cao, C., Yu, J., Wang, Y.-Q., Ying, Y.-L. & Long, Y.-T. Driven translocation of polynucleotides through an aerolysin nanopore. *Anal. Chem.* **88**, 5046-5049 (2016).

7. Kim, Y., Cha, M., Choi, Y., Joo, H. & Lee, J. Electrokinetic separation of biomolecules through multiple nano-pores on membrane. *Chem. Phys. Lett.* **561-562**, 63-67 (2013).
