# Supplementary material for: Identification of tagged glycans with a protein nanopore
Source: Nat Commun. 2023 Mar 28;14:1737. doi: 10.1038/s41467-023-37348-5 (PMC10050315; doi:10.1038/s41467-023-37348-5)
Supplement: Supplementary file 3 — Reporting Summary [file 41467_2023_37348_MOESM3_ESM.pdf]

nature portfolio

Corresponding author(s): Guangyan Qing

Last updated by author(s): Mar 10, 2023

Reporting Summary

Nature Portfolio wishes to improve the reproducibility of the work that we publish. This form provides structure for consistency and transparency in reporting. For further information on Nature Portfolio policies, see our Editorial Policies and the Editorial Policy Checklist.

Statistics

For all statistical analyses, confirm that the following items are present in the figure legend, table legend, main text, or Methods section.

n/a	Confirmed	
☐	☒	The exact sample size (*n*) for each experimental group/condition, given as a discrete number and unit of measurement
☐	☒	A statement on whether measurements were taken from distinct samples or whether the same sample was measured repeatedly
☒	☐	The statistical test(s) used AND whether they are one- or two-sided<br>*Only common tests should be described solely by name; describe more complex techniques in the Methods section.*
☒	☐	A description of all covariates tested
☒	☐	A description of any assumptions or corrections, such as tests of normality and adjustment for multiple comparisons
☐	☒	A full description of the statistical parameters including central tendency (e.g. means) or other basic estimates (e.g. regression coefficient) AND variation (e.g. standard deviation) or associated estimates of uncertainty (e.g. confidence intervals)
☒	☐	For null hypothesis testing, the test statistic (e.g. *F*, *t*, *r*) with confidence intervals, effect sizes, degrees of freedom and *P* value noted<br>*Give P values as exact values whenever suitable.*
☒	☐	For Bayesian analysis, information on the choice of priors and Markov chain Monte Carlo settings
☒	☐	For hierarchical and complex designs, identification of the appropriate level for tests and full reporting of outcomes
☒	☐	Estimates of effect sizes (e.g. Cohen's *d*, Pearson's *r*), indicating how they were calculated

Our web collection on statistics for biologists contains articles on many of the points above.

Software and code

Policy information about availability of computer code

Data collection	Data presented was generated experimentally in our lab and not collected. Nanopore current traces were recorded by Axopatch 200B applifier and Axon Digidata® 1550B Data Acquisition System and the accompanied commercially available pCLAMP™ 11 Software.
Data analysis	The recorded current traces were processed by using MOSAIC v2.2 software (https://pages.nist.gov/mosaic/). The obtained BlockedCurrent and dwell time were plotted and processed by Gaussian fitting with OriginPro 2021. The weight prediction in mixture was done with a custom code "MIX-distribution" that is available at https://doi.org/10.5281/zenodo.7714328. Machine learning was based a custom code "Glycan-Classification" that is available at https://doi.org/10.5281/zenodo.7711079. The molecular docking was performed based on the Schrödinger software suite (2022) (Schrödinger, LLC, New York, NY).

For manuscripts utilizing custom algorithms or software that are central to the research but not yet described in published literature, software must be made available to editors and reviewers. We strongly encourage code deposition in a community repository (e.g. GitHub). See the Nature Portfolio guidelines for submitting code & software for further information.

Data

Policy information about availability of data

All manuscripts must include a data availability statement. This statement should provide the following information, where applicable:

- Accession codes, unique identifiers, or web links for publicly available datasets
- A description of any restrictions on data availability
- For clinical datasets or third party data, please ensure that the statement adheres to our policy

The main data supporting the findings of this study are available within the main text, the Supplementary Information file, and the Source Data files. Additional raw data are available at https://doi.org/10.6084/m9.figshare.22241326.v2. Source data are provided with this paper.

Human research participants

Policy information about studies involving human research participants and Sex and Gender in Research.

Reporting on sex and gender	N/A
Population characteristics	N/A
Recruitment	N/A
Ethics oversight	N/A

Note that full information on the approval of the study protocol must also be provided in the manuscript.

Field-specific reporting

Please select the one below that is the best fit for your research. If you are not sure, read the appropriate sections before making your selection.

[x] Life sciences [ ] Behavioural & social sciences [ ] Ecological, evolutionary & environmental sciences

For a reference copy of the document with all sections, see nature.com/documents/nr-reporting-summary-flat.pdf

Life sciences study design

All studies must disclose on these points even when the disclosure is negative.

Sample size	Nanopore ionic current traces were recorded for enough long time to provide as least a total blockage events of 9,000 events to draw the scatter plot and histogram to fully display the population characteristic. Current traces containing too few events were repeated to reach this minimum statistical size.
Data exclusions	The extracted blockage events with Ib/I0 greater than0.99 (Ib/I0 value of equal to 1 indicates that the nanopore is completely unoccupied) were excluded from the statistics for the accurate representation of blockage signal. No further data was excluded from analyses.
Replication	The experiment of nanopore recording towards a glycan derivative was repeated several times. And the experiment is always successful as long as the assembled nanopore is stable within one to two hours.
Randomization	Randomization was not applicable to this study because we were not comparing experimental groups.
Blinding	Blinding was not applicable because there was no group allocation involved in this study.

Reporting for specific materials, systems and methods

We require information from authors about some types of materials, experimental systems and methods used in many studies. Here, indicate whether each material, system or method listed is relevant to your study. If you are not sure if a list item applies to your research, read the appropriate section before selecting a response.

Materials & experimental systems

n/a	Involved in the study
☒	☐ Antibodies
☒	☐ Eukaryotic cell lines
☒	☐ Palaeontology and archaeology
☒	☐ Animals and other organisms
☒	☐ Clinical data
☒	☐ Dual use research of concern

Methods

n/a	Involved in the study
☒	☐ ChIP-seq
☒	☐ Flow cytometry
☒	☐ MRI-based neuroimaging
